# Provenance Variation in Functional Traits of European Forest Trees: Meta‐Analysis Reveals Effects of Taxa and Age Despite Critical Research Gaps

**DOI:** 10.1002/ece3.71834

**Published:** 2025-08-06

**Authors:** Samuel Aspalter, Albert Ciceu, Carlos Miguel Landivar Albis, Debojyoti Chakraborty, Silvio Schueler

**Affiliations:** ^1^ Department of Forest Growth, Silviculture and Genetics Austrian Research Centre for Forests (BFW) Vienna Austria

**Keywords:** climate change adaptation, genecology, genetic diversity, intraspecific trait variation, provenance

## Abstract

Climate change is driving profound transformations in European forests. Understanding the adaptive potential of tree species is a key challenge for conservation and adaptation measures. A critical component of this adaptive potential lies in the intraspecific variation of functional traits. The long tradition in ecological genetics has resulted in a plethora of studies across species, regions, age classes, and traits. Prior syntheses have rarely quantified trait‐specific patterns and their variation across taxa and tree age. We conducted a systematic literature search to examine intraspecific variation in natural European tree populations. We identified four approaches to study intraspecific variation (i.e., provenance effects, provenance environment interaction effects, clinal effects and transfer effects). For each approach, we compared their prevalence to show an effect while also accounting for species, species group and age. Our results found that intraspecific variation is common in European tree species, with tested traits showing significant provenance effects (73%), provenance–environment interaction effects (45%), linear clinal effects (30%) and linear transfer effects (38%). While growth traits were predominantly studied, several other traits showed higher frequencies of significant results. Specifically, reproduction, survival, phenology, plant morphology, plasticity, drought, and frost tolerance are highly relevant but still understudied in comparison to growth. Conifer species demonstrated a higher prevalence of intraspecific variation compared to broadleaves. Despite the research clearly focusing on young trials, older trials tended to show higher frequencies of effects in phenology, growth, plant morphology, and survival, suggesting accumulating environmental selection with growing tree age. Europe lacks essential information on intraspecific variation of tree species for the diversification, conservation, and adaptation of its forests, especially in southern and southeastern parts, where many species harbour high genetic diversity and are most vulnerable. The significant influence of age urges for a reanalysis, reestablishment, and maintenance of long‐term trials. These trials should consider species and environmental conditions relevant for future scenarios.

## Graphical abstract

This meta‐analysis on intraspecific variation in European trees studied the frequency of provenance variation, provenance–environment interaction, clinal variation along the climate of origin or transfer distance. While identifying trait group specific patterns of variation, research gaps have been identified in terms of age and geographical distribution.

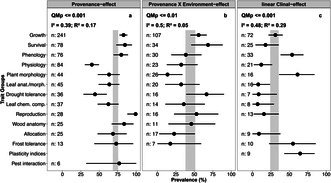

## 1 Introduction

Climate change is driving profound transformations in ecosystems globally, with forest ecosystems already exhibiting altered productivity, shifting species distributions, changes in economic value and heightened disturbance regimes such as insect outbreaks and extreme weather events (Allen et al. 2010; Chakraborty et al. 2021; Ciceu et al. 2024; Dyderski et al. 2018; Hanewinkel et al. 2013; Hlásny et al. 2021; Lindner et al. 2014; Mauri et al. 2022; Reyer et al. 2015; Seidl et al. 2011; Senf et al. 2018; Thom and Seidl 2016). Forest tree populations must respond to these pressures through adaptation, migration or face possible extinction (Aitken et al. 2008). However, long generation times, limited natural migration rates and increasing habitat fragmentation are likely to constrain evolutionary responses (Malcolm et al. 2002; Nathan et al. 2011; Sáenz‐Romero et al. 2016; Savolainen et al. 2007; Williams and Dumroese 2013), particularly in Europe, where glaciation history and biogeographic barriers have led to a relatively depauperate tree flora (Latham and Ricklefs 1993; Svenning and Skov 2007). As a result, understanding the adaptive potential of tree species is a key challenge for forest conservation and management (Nadeau and Urban 2019; Polechová 2018).

A critical component of this adaptive potential lies in the intraspecific variation of functional traits—traits that affect growth, survival, and reproduction (Violle et al. 2007). Variation within species is a result of phenotypic plasticity and local adaptation, often working antagonistically. In forest trees, such variation has long been studied in ecological genetics (formerly genecology) through provenance trials, which revealed significant genetic differentiation among populations, often reflecting local adaptation (Langlet 1971; Mátyás 1996). Traditionally, these studies focused on growth and timber‐relevant traits, which have also informed our understanding of climate adaptation and genetic diversity (Aitken and Bemmels 2016; Kapeller et al. 2017; Wang et al. 2010). However, growth traits only represent part of the picture. They are best seen as performance proxies rather than direct measures of evolutionary fitness. Other critical dimensions of fitness such as survival, reproduction, or stress tolerance are currently shown less attention.

**FIGURE 1:**
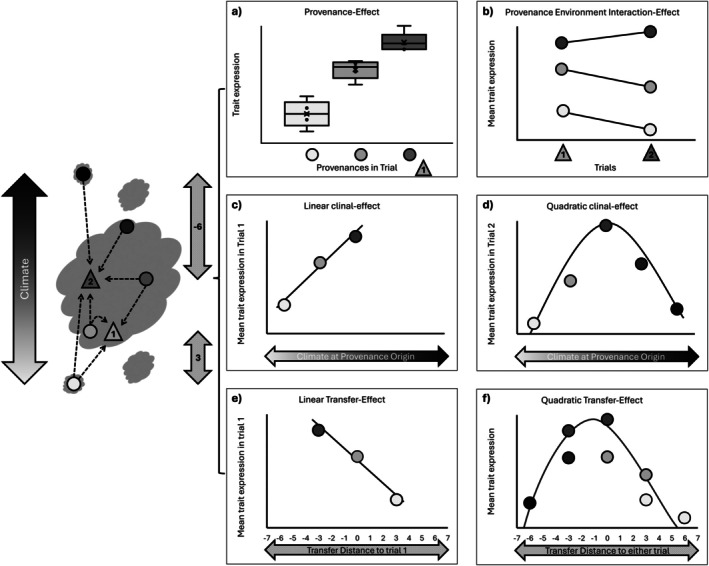
Different approaches to study intraspecific variation. Left of the panels is the distribution range of a fictive species along an environmental gradient and the transfer of five provenances to two trials in an unbalanced setup. The arrow on the right show examples of transfer distances (i.e., climate at provenance—climate at site) of a fictive climate variable for the northernmost provenance to the central trial and the southernmost provenance to the southern trial. The panels show several ways of analyzing the trials. (a) Significant differences in mean trait values of different provenances in one trial indicate *provenance effects*. (b) *Provenance–environment interaction* effects can be examined for the provenances that are tested at both sites. No interaction (i.e., parallel reaction, e.g., the two southern provenances) suggests that the pattern of intraspecific variation stays the same in different environments. Interaction means that there are provenance specific differences in plasticity (e.g., between central and southern provenances). We consider relations between climate at origin and provenance trait expressions as significant *clinal effects*. These can be (c) liner or (d) quadratic. Relations between transfer distance and trait expression describe *transfer effects*. (e) An individual transfer function for trial 1 which is linear in this case, but they are more commonly (f) quadratic and combine several trials.

Four approaches are commonly used to capture different aspects of intraspecific variation in such studies: provenance effects (PE; Figure 1a), indicating genetic differences among populations; provenance‐by‐environment interactions (P × E; Figure 1b), reflecting variation in phenotypic plasticity; clinal effects (CE; Figure 1c,d), where trait variation aligns with source‐climate gradients; and transfer effects (TE; Figure 1e,f), where trait variation aligns with environmental distance between source and planting site. While each of these has been modeled in various ways—often with overlapping methods, a meta‐analytical synthesis of their relative importance across functional traits remains scarce.

To add to this complexity, the long tradition in ecological genetics resulted in a plethora of studies across (tree) species and regions that use specimens of various ages and test a multitude of different traits. Numerous reviews already synthesized patterns of intraspecific variation and local adaptation in trees, each with its specific focus (Alberto et al. 2013; Leites and Benito Garzón 2023; Lind et al. 2018; Matesanz and Ramírez‐Valiente 2019; Park and Rodgers 2023; Ramírez‐Valiente et al. 2022). These efforts have significantly advanced our understanding of intraspecific variation and local adaptation. However, a systematic synthesis of intraspecific trait variation across all four concepts (PE, P × E, CE, TE) has not yet been conducted within a unified, trait‐centered, meta‐analytical framework. Prior syntheses have rarely quantified how trait‐specific patterns vary across taxa or tree age. We aim to complement and extend this body of work by providing a structured comparison of trait variation across these different sources of variation. Specifically, we aim to

1. Describe the current state of the field by assessing the number of studies per species, the geographic distribution of trials and provenances, and the age distribution of studies across trait groups, to identify biases and potential knowledge gaps.
2. Quantify and test the comparative importance of different functional traits for different sources of intraspecific variation, that is, provenance effects (PE), provenance‐by‐environment interactions (P × E), clinal effects (CE), and transfer effects (TE).
3. Test and compare patterns in functional traits between species groups (broadleaves vs. conifers), individual species, and age.

These objectives will support us in answering the following hypotheses: First, critical imbalance in terms of tree species and regions has been reported in other studies (Leites and Benito Garzón 2023; Park and Rodgers 2023). For Europe, we expect this to be the same here with pronounced research imbalance toward economically important tree species from central and northern Europe. Second, growth traits are usually dominant in reports of intraspecific variation (Matesanz and Ramírez‐Valiente 2019). However, other traits are ecologically even more important. We assume that functional traits more closely linked to fitness outcomes, such as survival, reproductive output, and stress tolerance, show a higher probability of expressing intraspecific variation than growth or other traits. Third, many studies report higher tendencies for intraspecific variation of coniferous species compared to broadleaved species. We assume that this will also be the case across trait groups. Fourth, although a large share of selection of forest trees takes place at juvenile stages due to competition, environmental pressures will only accumulate over time. Therefore, we assume that studies at old age are more likely to obtain significant trait variation.

Our study adds a novel quantitative synthesis to the field by bridging the gap between functional trait ecology and ecological genetics in European forest trees. By identifying underrepresented taxa, regions, traits, and study conditions, we highlight where knowledge is currently concentrated—and where it is lacking. By examining trait‐level responses across different modeling approaches and ages, we try to highlight the importance of life‐history traits other than growth and the importance of accumulated selection pressure in older trials.

## 2 Materials and Method

### 2.1 Literature Search, Selection and Extraction, Structure and Harmonization

We conducted a systematic literature search of scientific articles on the 88 European tree species that are present in the European Forest Genetic Resource Program using the websites Web of Science and Scopus, We used the scientific and the botanical English names of European tree species in combination with the search terms: “local adaptation” OR “genetic heterogeneity” OR “intraspecific variation” OR “population genomics” OR “genecolog*” OR “(provenance AND clima* AND respon*).” The query included searches in title, abstract, keywords (i.e., author keywords and keywords plus in WOK). It was conducted on February 2, 2022, with no date range applied (Appendix A).

**FIGURE D1:**
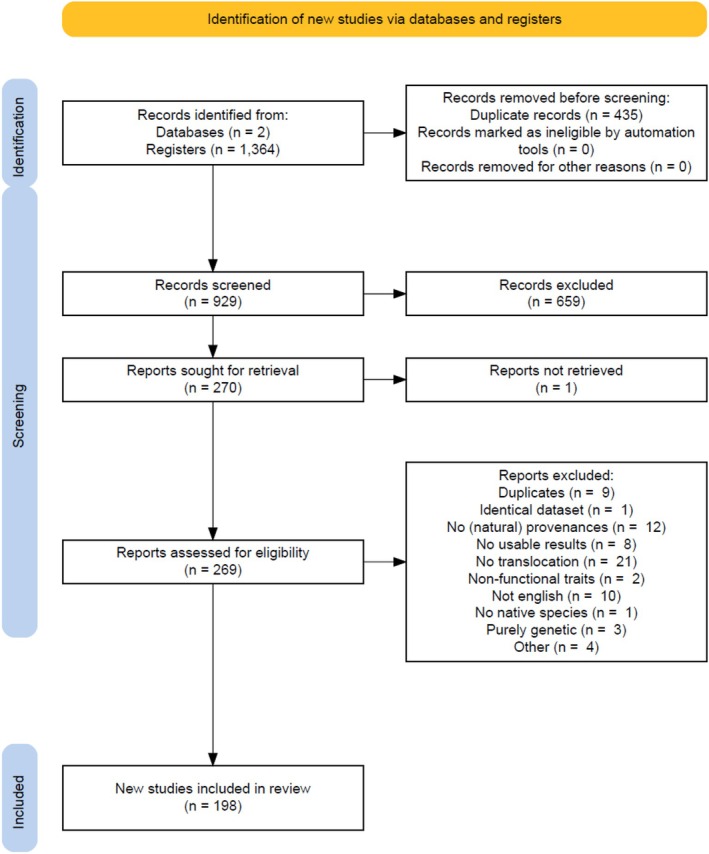
Flow diagram based on Haddaway (2022) showing consecutive reduction of articles. Citation: Haddaway, N. R., Page, M. J., Pritchard, C. C., & McGuinness, L. A. (2022). PRISMA2020: An R package and Shiny app for producing PRISMA 2020‐compliant flow diagrams, with interactivity for optimized digital transparency and Open Synthesis. *Campbell Systematic Reviews*, *18*(2), e1230. https://doi.org/10.1002/cl2.1230.

Based on title and abstract, we selected 269 articles that were further examined for extraction by two reviewers (Appendix A for a flow diagram (Figure D1) and selection criteria list). If an article examined several species, we treated each species as a distinct case (see also Matesanz and Ramírez‐Valiente (2019)). Additionally, articles were split into distinct cases if multiple methods (e.g., common garden and growth chamber experiment), or statistical analyses (e.g., ANOVA and Correlation) were used. For instance, if an article focused on a single species and a single trait, such as height, within one experimental design, like a common garden, it was considered one case. However, if the study analyzed height and provided separate, appropriate outputs for correlation and regression against climate at origin, we considered these two distinct cases. Similarly, if the article focused on a single trait but investigated multiple species, each species was treated as a separate case. Articles that proved to be not relevant or turned out to be duplicates initially not identified were subsequently excluded from the list and the reason for exclusion noted. We extracted 13 variables detailing study metadata (Table A1). In addition, we extracted the trait that was studied, the effect that was evaluated (i.e., PE, P × E, CE variables or TE variables) and whether this effect was significant or not.

A total of 915 distinct cases were extracted from the 198 investigated articles. Most articles evaluated intraspecific variation using categorical tests (like ANOVA and *t*‐test) leading to 361 cases for PE, of which 160 cases included interactions with environmental site conditions (Table 1) and therefore tested for P × E. We did not differentiate between the specific testing conditions, since the overall goal was to examine the general tendency for P × E. Accounting for the various testing environments while also keeping track of species and age classes would have resulted in a very imbalanced dataset. Likewise, although certainly of interest, we did not consider clinal variation against site conditions (so called response functions) as this would have clearly exceeded the scope of this review. Some of the tests did not examine PE explicitly but tested for range of origin (e.g., marginal or central population), climatic group, or country of origin. These were considered provenances by extension and included. Results for families or clones were not included in any analysis. CE was predominantly tested via linear regression, followed by correlations and quadratic models (134, 120, and 22 cases, respectively). In contrast, TE included predominantly quadratic models, followed by linear regressions and correlations against climate at origin (42, 36, and 20 cases, respectively). Finally, 25 cases studied CE or TE but did not use the analyses mentioned above.

**TABLE 1:** Summary showing the total number of cases per effect type.

Effect	Analysis	Cases
Provenance–effect	—	361
Provenance environment interaction‐effect	—	160
Clinal‐effect	Correlation	115
	Linear regression	134
	Quadratic regression	22
	Other	19
Transfer‐effect	Correlation	20
	Linear regression	36
	Quadratic regression	42
	Other	6

To enable a trait‐centered meta‐analysis, we harmonized and grouped the extracted data into defined functional trait categories. Specifically, we identified and grouped traits into 15 functional categories based on trait function, organ specificity, and their role in plant performance under environmental variation. While some of these categories (e.g., leaf anatomy, photosynthetic rate) represent classic functional traits sensu (Violle et al. 2007), others—such as survival, reproduction, and growth—are performance metrics or fitness‐related outcomes. We nonetheless included them in our framework because they were frequently reported in trait‐based studies, and they represent meaningful axes of plant response to environmental variation. This integrative approach allowed us to capture a broad spectrum of trait expression across studies.

*Allocation* represents the distribution of biomass or other resources within the plant (e.g., root‐to‐shoot ratio), reflecting internal investment strategies.

*Drought tolerance and frost tolerance* refer to plant responses to specific stress treatments, such as visible damage or differences in growth between control and stress conditions. By distinguishing these two abiotic stressors and focusing on treatment–control comparisons, we capture the direct tolerance capacity of genotypes. When trait values were reported under environmental treatments without a control comparison (e.g., height in drought conditions), we considered the treatment as background environment and assigned traits to their corresponding performance category—typically growth.

*Growth* encompasses quantitative measures of biomass accumulation or dimensional expansion (e.g., height, diameter, biomass), regardless of plant organ. Although it is often considered a performance outcome rather than a classic functional trait, we include it here due to its widespread use as a proxy for plant vigor and environmental response.

*Leaf morphology and leaf anatomy* were combined into one group, reflecting the common challenge of separating external and internal structural features.

Traits related to *chemical composition* were grouped by plant organ—leaf, stem, or root—rather than by compound type, as many compounds serve multiple physiological and ecological functions and were not always interpreted consistently across studies.

*Phenology* refers to seasonal developmental events such as budburst, leaf senescence, or growing season duration.

*Physiology* covers fundamental biochemical and biophysical processes (e.g., photosynthetic rate, stomatal conductance).

*Plant–animal interaction* captures biotic responses such as herbivory or pest infestation, which—though less frequently reported—represents important ecological dimensions of fitness.

*Plant morphology* refers to overall structural characteristics (e.g., stem straightness, tree form) distinct from individual organ growth.

*Plasticity indices* were treated as a separate category, as they quantify the variation in trait expression across environments and therefore reflect phenotypic flexibility rather than mean trait performance.

*Reproduction* includes any measure of reproductive success, such as quantity of flowering, fruiting, or seed germination. Like survival and growth, reproduction is often treated as a fitness component, but we include it due to its central role in life‐history strategies and environmental adaptation.

*Survival* includes both survival rates and mortality counts, which serve as direct indicators of fitness under experimental conditions. While not a functional trait in the narrow sense, survival represents a direct fitness component and is therefore included.

*Wood anatomy* includes structural properties of wood (e.g., density, vessel diameter), often linked to mechanical support or hydraulic function.

Traits that could not be clearly assigned to any of the above categories were grouped into an *Other* category and removed from the analyses.

The full list of individual traits and their respective group assignments is available in Appendix B. This categorization allowed for consistent comparison across studies and supports the identification of trait‐specific patterns of intraspecific variation across multiple dimensions of environmental response and adaptation. Our grouping decisions balanced biological relevance with the practical need for comparability across studies reporting heterogeneous trait data and reflect both ecological function and methodological consistency.

### 2.2 Data Analysis

#### 2.2.1 General Coverage of Species, Geography, Traits and Age

We counted the number of articles per species, the number of articles that referred to a specific country as test location and the number of articles that referred to a specific country as provenance location. The age distribution of all extracted observations was examined for the trait groups while also differentiating between conifers and broadleaves.

#### 2.2.2 Prevalence of Effect Types Among Traits Groups

**FIGURE D2:**
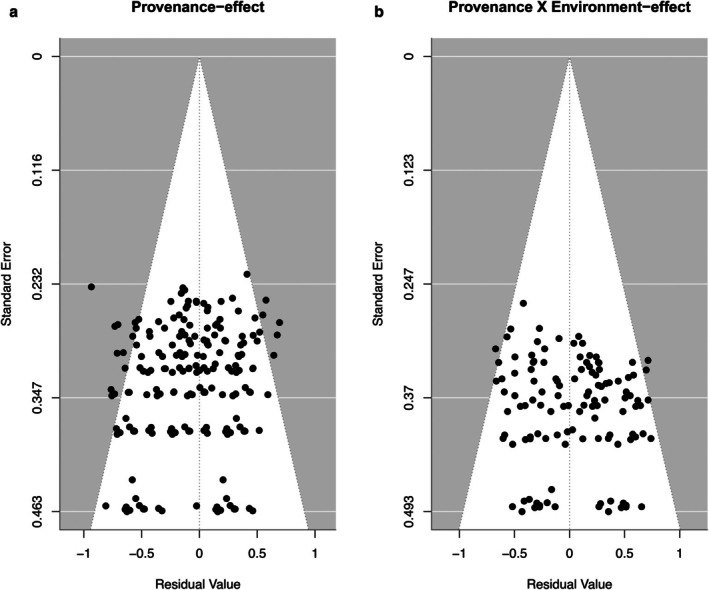
Funnel plot for the meta‐analysis of provenance effects (a) and provenance–enviornment interaction effects (b).

**FIGURE D3:**
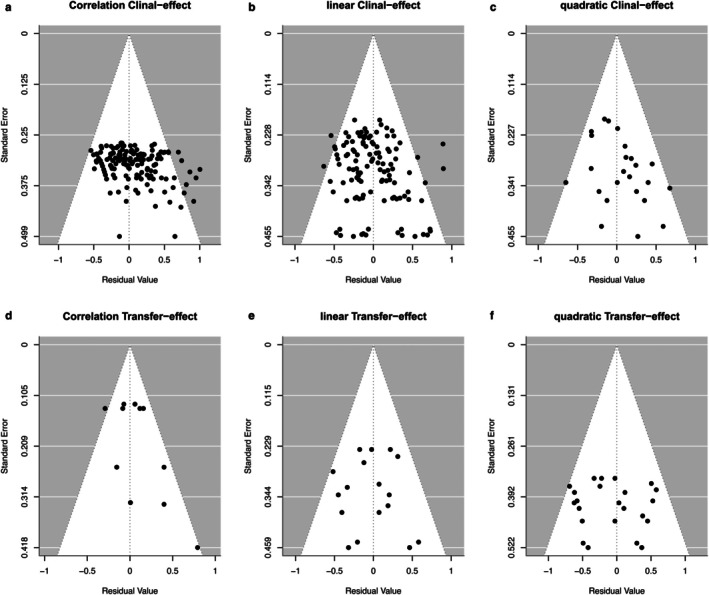
Funnel plot for the meta‐analysis of clinal (a–c) or transfer effects (d–f) using either correlation (a and d), regression with linear terms (b and e), or regression with quadratic terms (c and f).

We first aimed at identifying relevant trait groups that show strong trends for PE, P × E, CE, and TE. As mentioned in the introduction, a plethora of different methods was used to study intraspecific variation. However, the number of cases seemed extensive enough to describe overarching tendencies by calculating general prevalences for the different types of effects. Following established methods (Matesanz and Ramírez‐Valiente 2019; Ramírez‐Valiente et al. 2022), we divided the number of significant traits by the total number of studied traits per case and trait group to estimate the effect size. We conducted random effect analyses of proportions (Barendregt et al. 2013) to estimate the prevalence for PE, P × E, CE, and TE using the rma() function of the metafor package in the statistical software R. REML was used for model fitting, and Knapp‐Hartung adjustment (Knapp and Hartung 2003) was used to estimate confidence intervals (test = “knha” in the rma() function). For CE and TE, this was done individually for each of the different methods (i.e., correlations, linear terms, quadratic terms). To estimate the prevalence of effects for each trait group and how the trait groups varied in prevalence among each other, we included trait groups as categorical moderators for a subgroup analysis (Borenstein and Higgins 2013) using the same model specifics as above (using the mods = argument in the rma() function). Trait groups were only considered for subgroup analysis if more than four articles were available. The original ratios were transformed via Freeman–Tukey Double arcsine transformation (Freeman and Tukey 1950) while also using the total number of studied traits as sample size correcting for imbalances. A test statistic for between‐study heterogeneity (*I* ^2^; Higgins and Thompson 2002) was included in the analysis to account for intercase variation. It is the ratio of true to total variance. Results with an *I* ^2^ < 50% were considered sufficiently homogeneous for interpretation (Ryan and Cochrane Consumers and Communication Review Group 2016), that is, the variance is being mainly described by sampling error but not true variance in the data. By splitting the analyses according to methods and consulting *I* ^2^ we ensured that only homogeneous studies were compared. Since the focus of the primary studies was mainly to compare different provenances and their expressions in specific traits but not in reporting a high number of traits that vary significantly, we assumed that no publication bias affected our meta‐analysis. This can be confirmed by the funnel plots (Figures D2 and D3) which do not show strong asymmetries. Nevertheless, we are aware that our results will only be able to show overall patterns or tendencies and should be interpreted with caution. In addition, the close agreement of our results with more quantitative comparisons (see Discussion) underlines the validity of our results.

#### 2.2.3 Effect of Taxa and Age on the Importance of Different Traits

After having shown significant differentiation of trait groups via subgroup analysis, we subset the data into these trait groups to test the importance of different sources of variation. For each trait group and type of effect (PE, P × E, CE and TE), we conducted mixed effect models (Borenstein and Higgins 2013) that separately tested the effects of species group (broadleaves/conifers), species, and age as fixed effects and each case as a random effect. Like the subgroup analysis on trait groups, this was conducted in R using the mods = argument in the rma function. We only included subgroups with more than four articles per group, and continuous moderators were only regressed if at least 10 articles were available.

All meta‐analyses were conducted with the metafor package (v3.8‐1; Viechtbauer 2010) for R (v4.4.1; R Core Team 2022). All results have been back‐transformed using the functions provided in the package. The results were pooled, and graphical representations were made using ggplot2 (v3.5.1; Wickham 2016). The extracted data as well as the R code for running the analyses are available in the dryad repository associated with this article.

## 3 Results

### 3.1 General Summary of Studies

**FIGURE D4:**
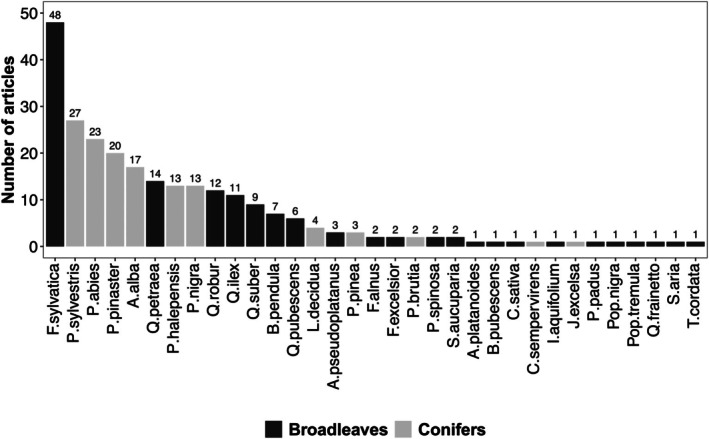
Bar chart with the frequency of studies per tree species.

We identified 198 articles published between 1974 and 2019 (A reference list of the data sources is found in Appendix E). We found information on 33 out of 88 target species. Among the 198 articles, 25 covered multiple species. The frequency of articles per species varied strongly, with the largest share of articles focusing on economically important, widely distributed, and mainly central European species (Figure D4). *Fagus sylvatica* (48) was the most studied species, followed by *Pinus sylvestris* , *Picea abies* , and *Pinus pinaster* (20–27 articles). A fair number of studies (11–16) have investigated *Abies alba* , *Quercus petraea* , *Pinus nigra* , *P. halepensis* , *Quercus robur* , and *Quercus ilex* , whereas only 4–9 articles were found for *Quercus suber* , *Betula pendula* , *Quercus pubescens*, and *Larix decidua* . Out of the total number of species studied, 19 were poorly represented, with only 1–3 studies per species (Figure D4).

**FIGURE 2:**
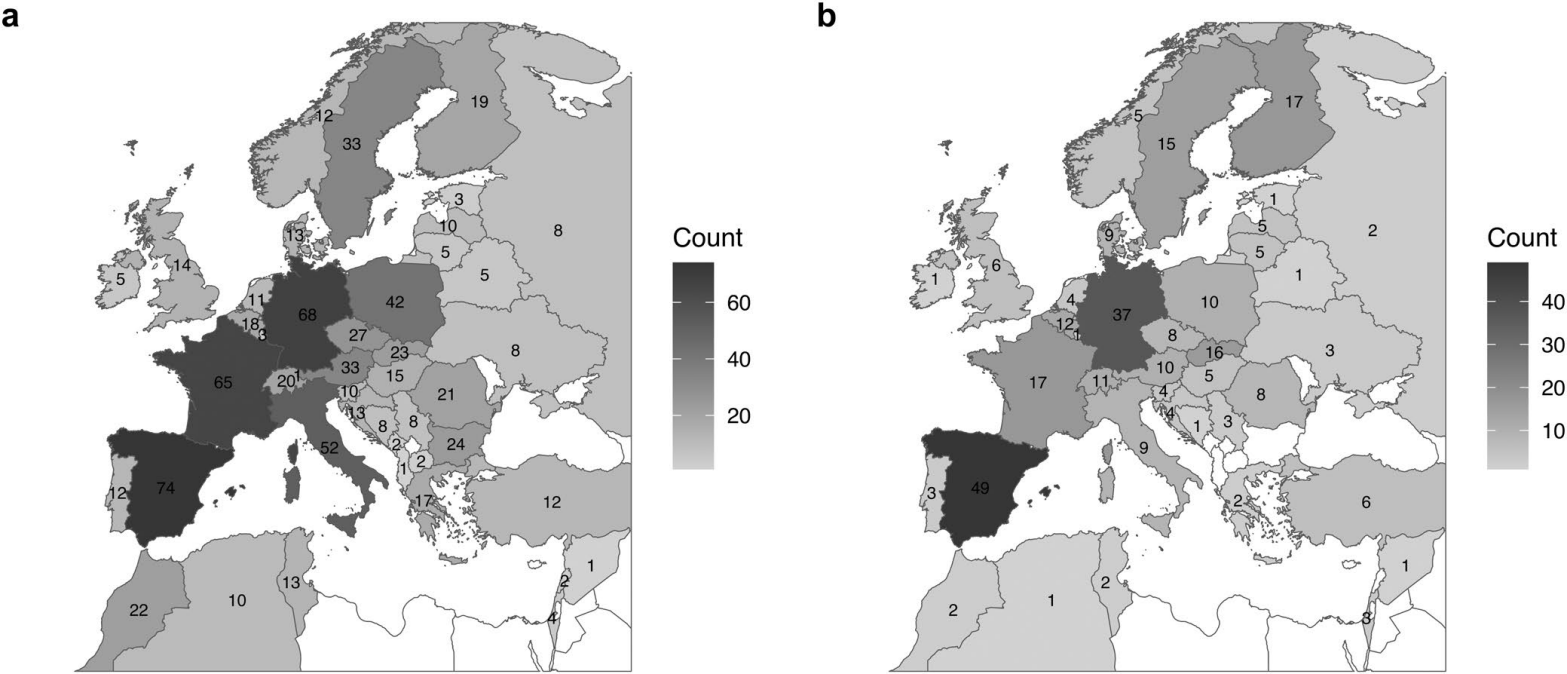
Maps showing the frequency of provenance testing (i.e., number of articles) by country (a) and showing the frequency of test implementation (i.e., number of articles) by country (b).

The coverage of provenance origins and study sites varied largely across Europe (Figure 2). Generally, provenances from southwestern and central Europe were predominantly tested, whereas the use of provenances from the Balkan and Baltic regions was limited (Figure 2a). Three countries, specifically Spain, Germany, and France, emerged as the primary sources for tested provenances, with 74, 68, and 65 articles, respectively. Here, the main species for provenance sampling were *Pinus pinaster* , with 18 and 17 articles for Spain and France and *Fagus sylvatica* , with 39 articles for Germany. In contrast, 16 countries only had less than 10 articles referring to them as the source of provenances (Figure 2a). Similarly, many articles conducted tests in Spain (49) and Germany (37) while articles with tests in eastern and southeastern Europe are underrepresented (Figure 2b). Countries that were sourced for provenances but from where no trials have been reported are Albania (1), Bulgaria (24), Lebanon (2), Liechtenstein (1), Montenegro (2) and North Macedonia (2). One third of the articles (60) were regional, being entirely based in one country. Conversely, 132 articles were transnational.

**FIGURE 3:**
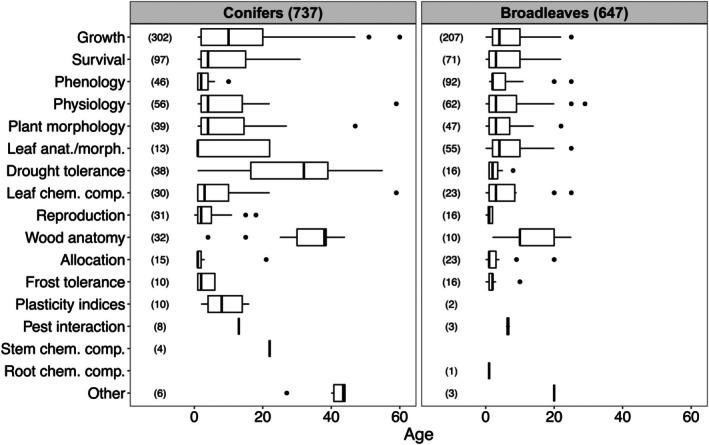
Age distribution within all studies by trait group separated for conifer and broad‐leafed species. The number in parentheses gives the number of cases. Trait groups with less than four studies were removed.

Most studies (99) examined seedlings (1–5 years) followed by 74 studies on juvenile trees (6–20 years), while only 29 studies focused on trees older than 20 years. Thus, when looking at the trait groups investigated, the majority were conducted on seedlings (11/16). Only Pest interaction and Plasticity indices were exclusively studied at juvenile ages. The general tendency is that—if at all—older stands were studied in conifers but not broadleaves. For example, only drought tolerance and wood anatomy (i.e., mainly analysis of tree rings) in conifers were investigated in older stands above 20 years (Figure 3). For broadleaves, drought tolerance and wood anatomy were studied at seedling and juvenile ages, respectively. Likewise, although overall growth was predominantly studied in seedlings, it was on average more often studied at higher ages in conifers than broadleaves.

### 3.2 Trait Groups With Prevalence for Intraspecific Variation

**FIGURE 4:**
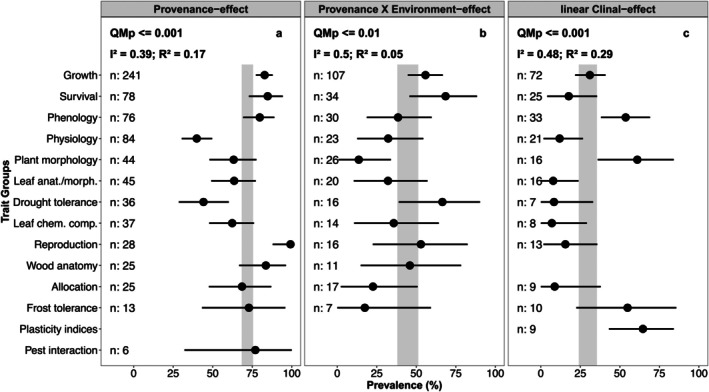
Significant results of the trait group subgroup analyses for the prevalence of showing (a) provenance effects, (b) provenance–environment interaction effects, and (c) linear clinal effects (see Figure D5 for the results in other methods). Points give the estimates for each trait group and lines the respective 95% confidence intervals. Gray bars give confidence intervals for the overall pooled effects. *N* gives the number of cases examined per trait group. QMp gives the *p* value for the *Q*‐test of subgroup differentiation. *R* ^2^ gives the variability accounted for by the subgroups. All analyses depicted had sufficient between‐case homogeneity (*I* ^2^ < 0.5).

The overall PE prevalence across all studies was 72% (Figure 4a). The test for subgroup differences was significant, and trait groups explained 17% of the variance in PE prevalence. Reproduction, wood anatomy, survival, growth, and phenology showed especially high prevalences, being on average higher than the overall estimate. Drought tolerance and physiological traits were the only groups that showed very little prevalence, whereas plant morphology, leaf morphology and leaf chemical composition showed moderate prevalence but were lower than the overall estimate. The estimates for allocation, frost tolerance and pest interaction had broad confidence intervals and were similar to the overall estimate.

The overall P × E prevalence was 45% (Figure 4b). The test for subgroup differences was significant, and trait groups explained 5% of the variance. Most traits showed very little prevalence for P × E. Only growth, survival, drought tolerance and reproduction were on average higher than the overall estimate. Many trait groups had broad confidence intervals for their estimate.

**FIGURE D5:**
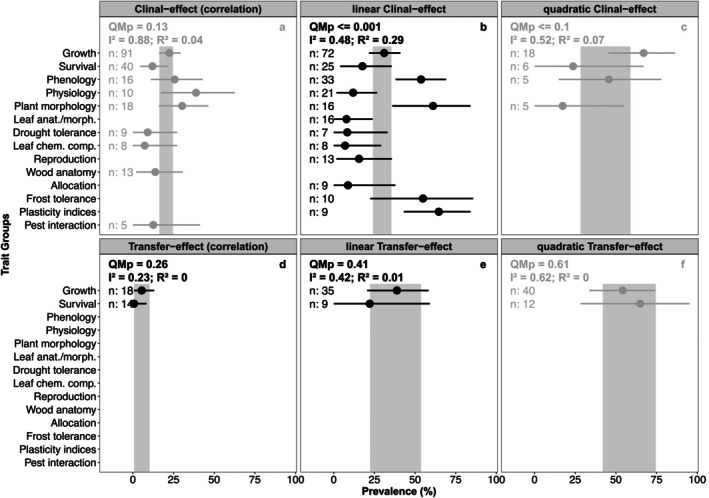
Results of the trait group subgroup analyses for the prevalence of showing significant clinal (a–c) or transfer effects (d–f). Both approaches include tests via correlations (a, d), regressions with linear terms (b, e) and regressions with quadratic terms (d, f). Points give the estimates for each trait group and lines the respective 95% confidence intervals. Gray bars give confidence intervals for the overall pooled effects. N gives the number of cases examined per trait group. QMp gives the *p* value for the *Q*‐test of subgroup differentiation. *R* ^2^ gives the variability accounted for by the subgroups. *I* ^2^ is an indicator of between study heterogeneity. If an analysis resulted in an *I* ^2^ higher than 0.5, it was grayed out.

For CE only, linear relationships showed significant variation with traits while also having low in‐between‐case heterogeneity compared to regressions and quadratic relationships (Figures 4c and D5 for comparison). The overall prevalence was 30% and the subgroup analysis of trait groups could explain 29% of the variance. Phenology, plant morphology, and frost tolerance traits showed prevalence, albeit the latter having high variability. Growth traits had a prevalence similar to the overall estimate. Quadratic CE only showed marginally significant variation (QMp ≤ 0.1) with traits explaining 7% of the variance but also showed relatively broad confidence intervals for the estimates (Figure D5c).

The general coverage of studies using TE was low and only growth and survival traits provided enough studies for comparison. Within‐study heterogeneity (*I* ^2^) was sufficiently low for studies using correlation and linear relationships (Figure D5d–f). However, no significant difference between growth and survival in TE prevalences was found. Studies on TE that used correlation as a method reported low prevalences (4%). Linear relationships showed a moderate overall estimate (38%) but broad confidence intervals for the estimate.

### 3.3 Effect of Taxa and Age

**FIGURE 5:**
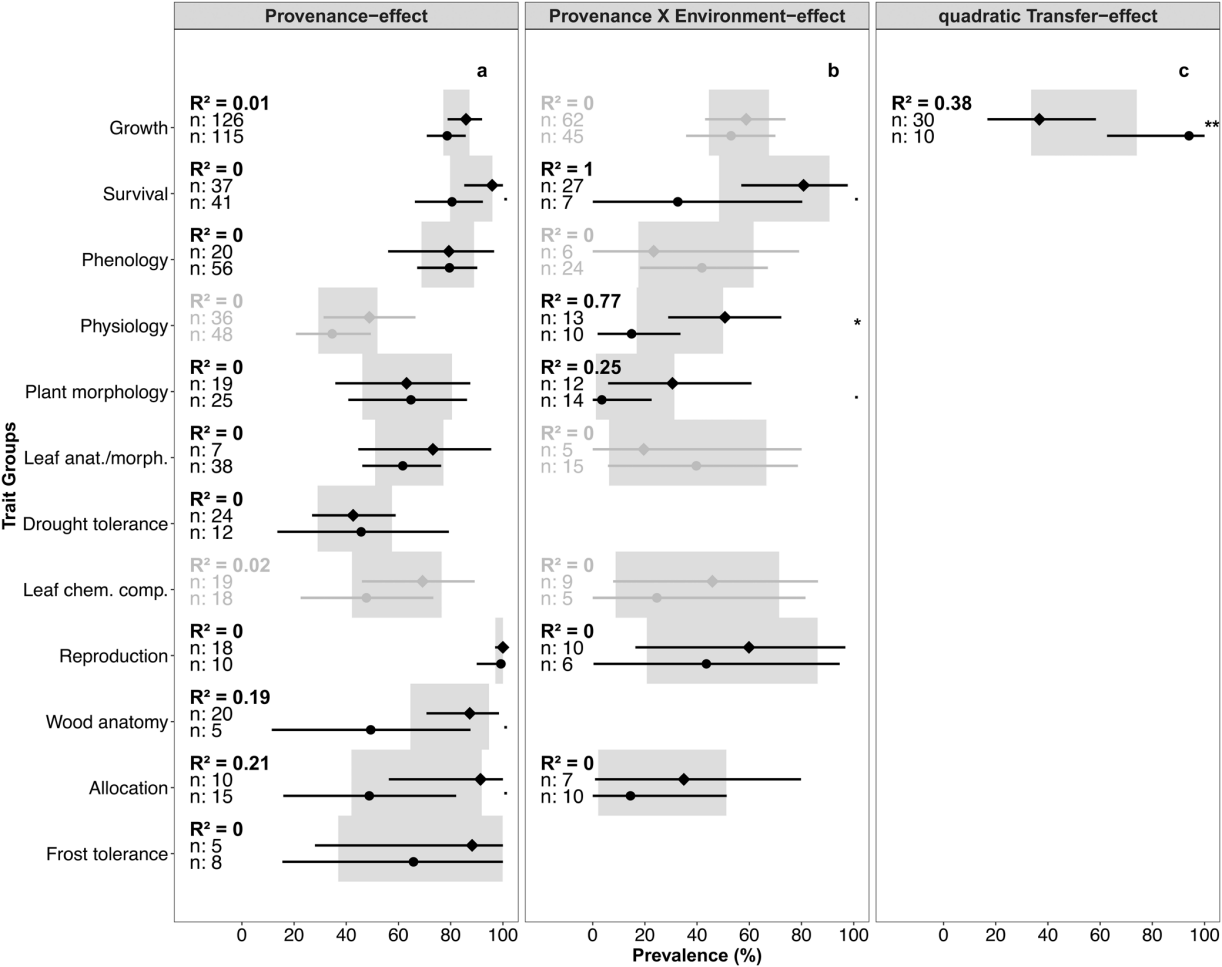
Results of the species group subgroup analyses within trait groups for the prevalence of showing significant (a) provenance, (b) provenance environment interaction, and (c) clinal effects. The latter only consists of cases using quadratic terms when regressing traits against transfer distance (see Figure D6 for the results in other methods). Diamonds and points give the estimate for conifers and broadleaves respectively and lines the respective 95% confidence intervals. Gray bars give confidence intervals for the overall pooled effects within the respective trait group. *N* gives the number of cases examined per species group within trait group. *R* ^2^ gives the variability accounted for by the species group. *I* ^2^ is an indicator of between study heterogeneity. If an analysis resulted in an *I* ^2^ higher than 0.5, it was grayed out. Significant and marginally significant Q‐tests of subgroup differentiation (QMp ≤ 0.01 ≤ 0.05 or ≤ 0.1) are indicated with a “**,” “*,” or “.” respectively.

**FIGURE D6:**
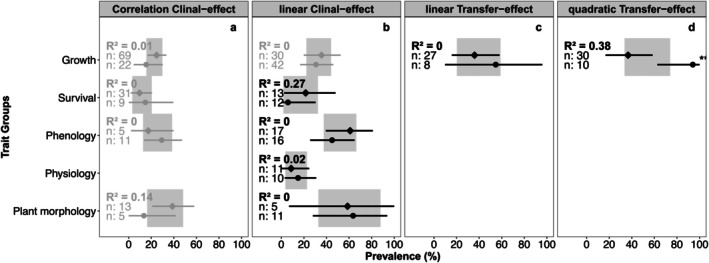
Results of the species group subgroup analyses within trait groups for the prevalence of showing significant clinal (a, b) or transfer effects (c, d). Not all effects allowed testing for all function types (correlation, regression including linear terms and regression including quadratic terms). Diamonds and points give the estimate for conifers and broadleaves, respectively, and lines the respective 95% confidence intervals. Gray bars give confidence intervals for the overall pooled effects within the respective trait group. N gives the number of cases examined per species group within trait group. *R* ^2^ gives the variability accounted for by the species group. *I* ^2^ is an indicator of between study heterogeneity. If an analysis resulted in an *I* ^2^ higher than 0.5, it was grayed out. Significant and marginally significant tests (*p* ≤ 0.01 ≤ 0.5 or ≤ 0.1) are indicated with a “**,” “*,” or “.” respectively.

A significant differentiation between broadleaved and conifer species in our models could be found in two incidences (Figure 5). For physiological traits in P × E, conifers had mean estimates of 51% compared to 15% for broadleaved species. Additionally, the prevalence for quadratic TE in growth traits was significantly different for broadleaved (94%) and coniferous species (37%; Figure D6). Marginally significant differentiation (QMp < = 0.1) was found for PE prevalence in survival, wood anatomy and allocation traits as well as for P × E prevalence in survival and plant morphology. In these incidents, conifers showed higher prevalences compared to broadleaved species.

Testing for individual species on the prevalence of significant effects was limited by the small overlap of studies examining the same trait groups between species. In total, nine analyses could be conducted for which enough studies per species and traits were available (Table C1). Of these, two incidences revealed significant differences between species while also showing acceptable low within‐case heterogeneity (*I* ^2^). Species explained 19% of variation in the PE prevalence in growth traits. Here, *Abies alba, Betula pendula, Picea abies, Pinus pinaster*, *Quercus petraea*, and *Quercus suber* showed higher provenance‐effect prevalence compared to the overall pooled average (80%), whereas *Fagus sylvatica* , *Pinus nigra* , and *Quercus ilex* showed lower prevalences, and *Pinus sylvestris* and *P. halepensis* prevalences close to the overall pooled average (Table C1). Additionally, species explained 50% of variation in PE prevalence in leaf chemical composition, with *Fagus sylvatica* having higher and *Quercus suber* lower prevalence compared to the overall pooled average (53%).

**FIGURE 6:**
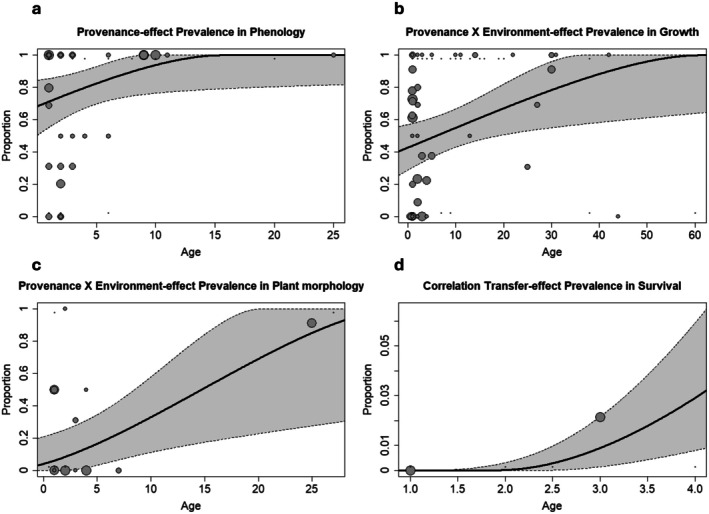
Bubble plots for significant results of meta‐regressions against age for (a) provenance–effect in phenological traits, (b) provenance–environment interaction effects in growth traits, (c) provenance–environment interaction effects in morphological traits, and (d) clinal effects in survival. Bubbles give the weighted estimates of individual cases. The line gives the backtransformed regression lines of the models with corresponding confidence interval bounds in gray. All regression results including nonsignificant ones can be seen in Table C2.

In the meta‐regression, we regressed the transformed effect sizes against the age of the specimens studied in the articles. Like in the subgroup analyses for species, the data were limited due to small overlaps and few studies being conducted at higher ages (Table C2). Nevertheless, age had a significant effect on PE prevalence in phenological traits and explained 17% of the total variance (Figure 6a), indicating an increasing prevalence of PE with age. Additionally, P × E prevalence was significant in growth and plant morphology traits (*R* ^2^ = 13% and 44%, Figure 6b,c respectively). Survival TE prevalence was also regressed significantly with age but showed low *R* ^2^ (Figure 6d).

## 4 Discussion

Our meta‐analysis revealed pronounced biases in species coverage, geographic range, and age‐class representation across the available literature. Quantitative trait data were retrievable for only 33 of the 88 European tree species assessed. Studies from southeastern Europe (a key hotspot of genetic diversity) were especially scarce, leaving more than 60% of species and extensive portions of their native ranges without empirical evidence. In addition, patterns of intraspecific variation differed markedly among traits, taxa and age classes, underscoring the need for broader and more balanced sampling.

### 4.1 Gaps in Current Knowledge

Our meta‐analysis exposes pronounced taxonomic, geographic, and ontogenetic biases in the European tree‐trait literature. As hypothesized, more than half of the available studies (62%, *n* = 123) focused on European beech ( *Fagus sylvatica* ) and four commercially important conifers—Scots pine ( *Pinus sylvestris* ), Norway spruce ( *Picea abies* ), maritime pine ( *Pinus pinaster* ), and silver fir ( *Abies alba* )—while the remaining 83 species received comparatively scant attention. Research effort was likewise concentrated on central‐European and western‐Mediterranean countries (Germany, France, Spain, and Italy), leaving south‐eastern Europe—a hotspot of genetic diversity—under‐represented. A similar conifer‐centered bias has been documented for North America (Park and Rodgers 2023) and at the global scale (Leites and Benito Garzón 2023).

The prevailing emphasis on central‐ and western‐European provenances leaves a critical knowledge gap for southeastern Europe—one of the continent's principal reservoirs of genetic diversity. Phylogeographic studies show that the Balkans hosted several of the largest Pleistocene glacial refugia, preserving deep evolutionary lineages and exceptionally high allelic richness (Petit et al. 2003; Tzedakis 2004; Postolache et al. 2017). Consequently, Balkan populations often display not only greater molecular diversity but also distinct local adaptations, as demonstrated for silver fir (Bosela et al. 2016). Long‐term provenance trials established in this region therefore constitute a natural “time‐machine,” offering climate analogs for the conditions projected in central Europe later this century (Leimu and Fischer 2008; Matesanz and Ramírez‐Valiente 2019; Mette et al. 2021; Park and Rodgers 2023; Ramírez‐Valiente et al. 2022). Neglecting southeastern Europe thus risks overlooking key adaptive potential and misinforming assisted migration and conservation strategies for the wider continent.

Last, we admit that treating entire countries as analytical units is inherently coarse, because their size and geographic position distort their relevance for any individual species' range. Ideally, each provenance and common‐garden trial would be geo‐referenced along explicit environmental or genetic gradients. That level of detail would allow us to classify populations consistently—e.g., as xeric‐marginal, mesic‐core, or drought‐prone central—relative to the species' distribution. However, provenance‐ and trial‐specific metadata were reported sporadically and in incompatible formats, precluding a harmonized extraction of such information across studies.

### 4.2 Trait Groups With Prevalence for Intraspecific Variation

#### 4.2.1 Provenance Effect Prevalence

Across European tree species, intraspecific variation is the rule rather than the exception: provenance explained significant differences in 73% of the 738 observations. This aligns with the 90% prevalence of provenance effects (PE) reported by Alberto et al. (2013) for common‐garden trials and 83% reported by Ramírez‐Valiente et al. (2022) for Mediterranean but mainly European species. By contrast, a global synthesis of tree phenotypic plasticity found lower rates—54% indicating a greater within‐species variation of European compared to a worldwide average, albeit this study also reported a bias towards temperate tree species (Matesanz and Ramírez‐Valiente 2019).

The trait‐specific patterns emerging from our synthesis parallel the quantitative survey of population differentiation conducted by Alberto et al. (2013). Across 19 traits, they reported an overall weighted mean QST of 0.294, yet germination—a proxy for early‐life reproductive success—stood out with a mean QST of 0.52, echoing the strong PE we detected for reproductive traits. Growth traits (mean QST ≈0.318 across height and diameter) and phenological traits (mean QST ≈0.286 across budburst and leaf‐flush) exhibited only slightly above‐average differentiation, matching the intermediate levels in our meta‐analysis. By contrast, physiological traits showed minimal structuring (mean QST = 0.053), consistent with their low prevalence of PE in our data set. Frost hardiness displayed the broadest range of differentiation (QST = 0.126–0.581) and a higher‐than‐average mean (0.390), corroborating our finding that cold‐tolerance responses vary markedly among provenances.

Because provenance trials ultimately aim to identify seed sources with the highest fitness under target conditions, any trait measured functions as a fitness proxy. Growth metrics dominate the literature—as they do in our dataset—because, in mesic temperate forests, competition for light, water, and nutrients is assumed to be the primary selective pressure, and selection is expected to optimize growth via correlated functional traits. Yet that premise is unlikely to hold in regions where other stresses, such as drought or frost, govern survival. Accordingly, our synthesis shows that provenance effects are just as common—and in some cases, more widespread—in traits that are more directly tied to lifetime fitness, including reproduction, survival, wood‐anatomical properties, and phenology.

#### 4.2.2 Provenance × Environment Effect Prevalence

Phenotypic plasticity also varied among populations: 45% of the 321 observations we analyzed exhibited significant provenance‐by‐environment (P × E) interactions, indicating that different provenances responded to changes in environmental conditions in contrasting ways. Again, this prevalence closely matches the patterns reported for Mediterranean (mainly European) tree species, where Ramírez‐Valiente et al. (2022) found significant P × E in 53% of the traits assessed per study, while the worldwide assessment by Matesanz and Ramírez‐Valiente (2019) only reported 33%.

Among the lifetime fitness traits mentioned above—reproduction, survival, wood anatomy, phenology, and growth—survival displayed the strongest P × E signal, while reproduction and growth also exceeded the overall average, confirming that reaction norms differ markedly among provenances. Phenology, on the other hand, showed only limited P × E, indicating that while there is variation between provenances, this variation is likely to stay constant between changing environments. Contrastingly, drought tolerance, although seldom exhibiting PE, nevertheless showed pronounced P × E interactions, implying that drought response only shows intraspecific variation in phenotypic plasticity but not provenances directly.

#### 4.2.3 Clinal Effect Prevalence

Clinal effect (CE) prevalences were more common for phenology, plant morphology, frost tolerance and overall plasticity when linear relationships were studied. Presumably this is because these traits track broad latitudinal, longitudinal or elevational gradients that impose largely unidirectional selection. Growth metrics displayed only limited clinal effects (CE), mainly when quadratic terms were fitted. However, quadratic relationships in CE also showed heterogeneity (*I* ^2^) above our threshold. By contrast, both foliar trait groups showed below‐average prevalence for all effects (PE, P × E and CE), indicating that leaf attributes contribute less often to intraspecific adaptive divergence. This is plausible given the continual turnover of leaves and the pronounced within‐tree heterogeneity created by sun‐ and shade‐exposed foliage.

Across all trait–predictor combinations, linear clines were detected in only 30% of tests—substantially below the 78% reported by Alberto et al. (2013), who included studies that exclusively regressed trait variation against altitude, latitude and occasionally longitude and did not adjust for the number of climatic variables examined. Limiting our meta‐analysis to these three geographic predictors raised CE prevalence to 45% (results not shown) but still fell short of their estimate. Part of the discrepancy likely stems from study scope: our synthesis integrates both range‐wide and regional provenance trials to evaluate trait‐group patterns, whereas Alberto et al. (2013) considered only range‐wide data sets constructed for species‐level modeling.

#### 4.2.4 Transfer Effect Prevalence

No differences between survival and growth traits were found for transfer effect (TE) prevalences, which were also the only traits being reported in a sufficient amount for comparison. With only 4% TE prevalence, it was low when correlations were used. Contrastingly, linear relationships in TE were more common (38%) but this estimate showed little precision.

Overall, we see our second hypothesis confirmed that other life‐history traits than growth show equal and sometimes even more pronounced tendencies for intraspecific variation. With slight differences, this is the case across study approaches.

### 4.3 Effect of Taxa and Age

Subgroup analyses by taxonomic group and meta‐regressions incorporating age exposed substantial data gaps—many models could not be fitted because data sets were too sparse or heterogeneous. This rendered the testing of our third hypothesis more difficult. However, where evidence was sufficient, conifers consistently displayed higher PE prevalence than broadleaved taxa, mirroring patterns noted by Mátyás et al. (2019). Leites and Benito Garzón (2023) likewise reported significant intraspecific differentiation in 14 of 16 conifers (88%) but in only 8 of 12 broadleaved species (66%). In our analysis, this was particularly found for survival, physiology, plant morphology, wood anatomy and allocation but, surprisingly, not for growth. For growth, the strength of its provenance signal varied more among individual species than between the two taxonomic groups, and species group per se had only an effect for TE prevalence, where broadleaves showed higher mean prevalence than conifers when quadratic relationships were studied. Specifically, PE prevalence was significantly modulated by species for growth and leaf chemical composition.

The preponderance of provenance trials on juvenile trees largely reflects practical constraints and a desire to isolate abiotic adaptation: seedlings are raised at wide spacing to minimize biotic interactions, and experiments can be completed within conventional funding cycles. Again, this complicated testing of our fourth hypothesis. Yet our synthesis shows that some intraspecific variation signals intensify with stand age—especially for growth, phenology, plant morphology, and survival. Similar lag effects have been documented elsewhere: Germino et al. (2019) detected provenance‐based trait divergence only after ∼20 years in a North American shrub, whereas Franklin (1979) argued that genotypic selection in trees does not emerge until roughly half the rotation age, owing to differences between juvenile and mature performance. Two mechanisms likely underpin this pattern. First, older trials integrate a longer sequence of site conditions, including episodic stresses and extreme events, thereby exposing cryptic adaptation or maladaptation (Park and Rodgers 2023; Park and Talbot 2018). Second, the onset of intraspecific competition progressively filters genotypes, making density‐dependent interactions themselves a driver of adaptive divergence. These findings caution against a short‐term perspective: climate alone is an incomplete proxy for selective environments, and edaphic factors, competition and herbivory—often neglected in common‐garden designs—may be equally influential in shaping intraspecific variation.

## 5 Conclusion

The imbalance in provenance research leads to major research gaps. As highlighted in many reviews, other common and ecologically relevant species (such as from the genera *Acer, Fraxinus*, *Ulmus*, or Salix to name a few) are poorly studied (Park and Rodgers 2023), leading to a lack of information for the diversification of European forests and the required establishment of mixed stands. What is more, we currently miss information on species from southern and southeastern Europe, which are threatened even more by climate change since precipitation patterns change faster and many populations are fragmented (Alberto et al. 2013; Williams and Dumroese 2013). This geographic research gap, which is also reflected in the distribution of trials and provenances, needs to be filled to develop the foreknowledge needed for climate change adaptation. Therefore, we strongly recommend the establishment of new trials that also consider species and environmental conditions relevant for future scenarios (Matesanz and Ramírez‐Valiente 2019). The positive influence of age on the prevalence of intraspecific variation urges for a reanalysis, establishment and maintenance of long‐term trials as well as the substitutional use of inventories where possible (e.g., Fréjaville et al. 2019). When establishing new trials, they should be of such a size and management concept that allows for continued testing also when developed into old stands. As discussed, this age effect might also speak for more varied testing conditions that also include edaphic and biotic treatments in provenance tests (Park and Rodgers 2023; Park and Talbot 2018). Reproduction, frost and drought tolerance, survival, phenology and especially plasticity therein are valuable though still understudied traits for local adaptation and thus should be the subject of deeper investigation in future studies.

## Author Contributions

**Samuel Aspalter:** conceptualization (equal), data curation (lead), formal analysis (lead), investigation (lead), methodology (lead), software (lead), visualization (lead), writing – original draft (equal), writing – review and editing (equal). **Albert Ciceu:** data curation (supporting), formal analysis (supporting), investigation (supporting), methodology (supporting), software (supporting), writing – original draft (equal), writing – review and editing (equal). **Carlos Miguel Landivar Albis:** formal analysis (supporting), methodology (supporting), writing – original draft (equal), writing – review and editing (equal). **Debojyoti Chakraborty:** conceptualization (equal), funding acquisition (supporting), supervision (supporting), writing – original draft (equal), writing – review and editing (equal). **Silvio Schueler:** conceptualization (equal), funding acquisition (lead), investigation (supporting), methodology (supporting), supervision (lead), writing – original draft (equal), writing – review and editing (equal).

## Conflicts of Interest

The authors declare no conflicts of interest.

## Data Availability

The database and code compiled for this study are available on the Dryad repository: https://doi.org/10.5061/dryad.v15dv424v.

## Appendix A Literature Search and Extraction

### Search Terms

#### Web of Science (February 02, 2022)

(((TI = ((“ *Abies alba* ” OR “silver fir” OR “Abies borisii‐regis” OR “King Boris fir” OR “Abies bornmuelleriana” OR “Bornmuller's fir” OR “Bornmüller's fir” OR “Abies cephalonica” OR “Grecian fir” OR “Greek fir” OR “Abies nebrodensis” OR “Sicilian fir” OR “Abies pinsapo” OR “Spanish fir” OR “ *Acer campestre* ” OR “field maple” OR “hedge maple” OR “maser” OR “Acer monspessulanum” OR “French maple” OR “Montpellier maple” OR “ *Acer opalus* ” OR “Italian maple” OR “opalus maple” OR “ *Acer platanoides* ” OR “Norway maple” OR “ *Acer pseudoplatanus* ” OR “Sycamore” OR “ *Aesculus hippocastanum* ” OR “horse chestnut” OR “ *Alnus cordata* ” OR “Italian alder” OR “ *Alnus glutinosa* ” OR “black alder” OR “ *Alnus incana* ” OR “gray alder” OR “gray alder” OR “speckled alder” OR “ *Alnus viridis* ” OR “Green alder” OR “ *Betula pendula* ” OR “weeping birch” OR “warty birch” OR “ *Betula pubescens* ” OR “downy birch” OR “mountain birch” OR “silver birch” OR “swamp birch” OR “ *Carpinus betulus* ” OR “hornbeam” OR “ *Castanea sativa* ” OR “sweet chestnut” OR “Spanish chestnut” OR “ *Corylus avellana* ” OR “common hazel” OR “ *Corylus colurna* ” OR “Turkish hazel” OR “ *Celtis australis* ” OR “European nettle tree” OR “ *Cornus mas* ” OR “Cornelian cherry” OR “ *Cornus sanguinea* ” OR “Common dogwood” OR “ *Cupressus sempervirens* ” OR “Italian cypress” OR “Fagus orientalis” OR “Oriental beech” OR “ *Fagus sylvatica* ” OR “European beech” OR “Fagus taurica” OR “Crimean beech” OR “ *Frangula alnus* ” OR “Glossy buckthorn” OR “Fraxinus angustifolia” OR “narrow‐leafed ash” OR “ *Fraxinus excelsior* ” OR “european ash” OR “common ash” OR “ *Fraxinus ornus* ” OR “manna ash” OR “ *Ilex aquifolium* ” OR “Common holly” OR “ *Juglans regia* ” OR “walnut” OR “ *Juniperus communis* ” OR “Common juniper” OR “ *Juniperus excelsa* ” OR “Greek juniper” OR “ *Juniperus oxycedrus* ” OR “Prickly juniper” OR “ *Larix decidua* ” OR “European larch” OR “ *Malus sylvestris* ” OR “Wild apple” OR “Ostrya carpinifolia” OR “hop‐hornbeam” OR “hophornbeam” OR “ *Picea abies* ” OR “Norway spruce” OR “ *Picea omorika* ” OR “Serbian spruce” OR “ *Pinus brutia* ” OR “Brutia pine” OR “ *Pinus cembra* ” OR “Swiss stone pine” OR “ *Pinus halepensis* ” OR “Aleppo pine” OR “ *Pinus heldreichii* ” OR “Bosnian pine” OR “ *Pinus mugo* ” OR “Mountain pine” OR “ *Pinus nigra* ” OR “European black pine” OR “black pine” OR “ *Pinus peuce* ” OR “Macedonian pine” OR “ *Pinus pinaster* ” OR “Maritime pine” OR “ *Pinus pinea* ” OR “Stone pine” OR “ *Pinus sylvestris* ” OR “Scots pine” OR “ *Pinus uncinata* ” OR “Mountain pine” OR “ *Platanus orientalis* ” OR “Oriental plane” OR “ *Populus alba* ” OR “silver‐leaved poplar” OR “white poplar” OR “abell” OR “silver poplar” OR “silverleaf poplar” OR “silver‐leaf poplar” OR “silver leaved poplar” OR “ *Populus nigra* ” OR “black poplar” OR “ *Populus tremula* ” OR “European aspen” OR “trembling poplar” OR “ *Prunus avium* ” OR “sweet cherry” OR “bird cherry” OR “wild cherry” OR “ *Prunus cerasifera* ” OR “Cherry plum” OR “ *Prunus padus* ” OR “Bird cherry” OR “ *Prunus spinosa* ” OR “buckthorn” OR “ *Pyrus pyraster* ” OR “wild pear” OR “ *Quercus cerris* ” OR “Turkey oak” OR “Austrian oak” OR “Quercus coccifera” OR “Kermes oak” OR “ *Quercus frainetto* ” OR “Hungarian oak” OR “Italian oak” OR “ *Quercus ilex* ” OR “Holm oak” OR “Quercus pedunculiflora” OR “Grayish oak” OR “ *Quercus petraea* ” OR “durmast oak” OR “sessile oak” OR “cornish oak” OR “Quercus pubescens” OR “downy oak” OR “pubescent oak” OR “ *Quercus robur* ” OR “common oak” OR “english oak” OR “pedunculate oak” OR “ *Quercus suber* ” OR “Cork oak” OR “Quercus trojana” OR “Macedonian oak” OR “Quercus virgiliana” OR “Oak of Virgil” OR “ *Salix alba* ” OR “White willow” OR “ *Salix caprea* ” OR “Goat willow” OR “ *Sorbus aria* ” OR “whitebear” OR “beam tree” OR “chess apple” OR “ *Sorbus aucuparia* ” OR “rowan” OR “mountain ash” OR “ *Sorbus domestica* ” OR “service tree” OR “sorb tree” OR “whitty pear” OR “ *Sorbus intermedia* ” OR “Swedish whitebear” OR “ *Sorbus torminalis* ” OR “checker tree” OR “chequer tree” OR “wild service tree” OR “ *Taxus baccata* ” OR “yew” OR “ *Tilia cordata* ” OR ((“littleleaf” OR small*leaf OR small*leaved) AND (“lime” OR “linden”)) OR “ *Tilia platyphyllos* ” OR ((broad*leaved OR broad*leaf OR large*leaved OR large*leaf) AND (lime OR linden)) OR “ *Tilia tomentosa* ” OR “Silver lime” OR “Silver linden” OR “ *Ulmus carpinifolia* ” OR “ *Ulmus minor* ” OR “field elm” OR “small‐leaved elm” OR “ *Ulmus glabra* ” OR “Scots elm” OR “wych elm” OR “Ulmus laevis” OR “white elm” OR “fluttering elm” OR “Russian elm” OR “spreading elm”) AND (“local adaptation” OR “genetic heterogeneity” OR “intraspecific variation” OR genecolog* OR “population genomics” OR (provenance AND clima* AND respon*)))) OR AB = ((“ *Abies alba* ” OR “silver fir” OR “Abies borisii‐regis” OR “King Boris fir” OR “Abies bornmuelleriana” OR “Bornmuller's fir” OR “Bornmüller's fir” OR “Abies cephalonica” OR “Grecian fir” OR “Greek fir” OR “Abies nebrodensis” OR “Sicilian fir” OR “Abies pinsapo” OR “Spanish fir” OR “ *Acer campestre* ” OR “field maple” OR “hedge maple” OR “maser” OR “Acer monspessulanum” OR “French maple” OR “Montpellier maple” OR “ *Acer opalus* ” OR “Italian maple” OR “opalus maple” OR “ *Acer platanoides* ” OR “Norway maple” OR “ *Acer pseudoplatanus* ” OR “Sycamore” OR “ *Aesculus hippocastanum* ” OR “horse chestnut” OR “ *Alnus cordata* ” OR “Italian alder” OR “ *Alnus glutinosa* ” OR “black alder” OR “ *Alnus incana* ” OR “gray alder” OR “gray alder” OR “speckled alder” OR “ *Alnus viridis* ” OR “Green alder” OR “ *Betula pendula* ” OR “weeping birch” OR “warty birch” OR “ *Betula pubescens* ” OR “downy birch” OR “mountain birch” OR “silver birch” OR “swamp birch” OR “ *Carpinus betulus* ” OR “hornbeam” OR “ *Castanea sativa* ” OR “sweet chestnut” OR “Spanish chestnut” OR “ *Corylus avellana* ” OR “common hazel” OR “ *Corylus colurna* ” OR “Turkish hazel” OR “ *Celtis australis* ” OR “European nettle tree” OR “ *Cornus mas* ” OR “Cornelian cherry” OR “ *Cornus sanguinea* ” OR “Common dogwood” OR “ *Cupressus sempervirens* ” OR “Italian cypress” OR “Fagus orientalis” OR “Oriental beech” OR “ *Fagus sylvatica* ” OR “European beech” OR “Fagus taurica” OR “Crimean beech” OR “ *Frangula alnus* ” OR “Glossy buckthorn” OR “Fraxinus angustifolia” OR “narrow‐leafed ash” OR “ *Fraxinus excelsior* ” OR “european ash” OR “common ash” OR “ *Fraxinus ornus* ” OR “manna ash” OR “ *Ilex aquifolium* ” OR “Common holly” OR “ *Juglans regia* ” OR “walnut” OR “ *Juniperus communis* ” OR “Common juniper” OR “ *Juniperus excelsa* ” OR “Greek juniper” OR “ *Juniperus oxycedrus* ” OR “Prickly juniper” OR “ *Larix decidua* ” OR “European larch” OR “ *Malus sylvestris* ” OR “Wild apple” OR “Ostrya carpinifolia” OR “hop‐hornbeam” OR “hophornbeam” OR “ *Picea abies* ” OR “Norway spruce” OR “ *Picea omorika* ” OR “Serbian spruce” OR “ *Pinus brutia* ” OR “Brutia pine” OR “ *Pinus cembra* ” OR “Swiss stone pine” OR “ *Pinus halepensis* ” OR “Aleppo pine” OR “ *Pinus heldreichii* ” OR “Bosnian pine” OR “ *Pinus mugo* ” OR “Mountain pine” OR “ *Pinus nigra* ” OR “European black pine” OR “black pine” OR “ *Pinus peuce* ” OR “Macedonian pine” OR “ *Pinus pinaster* ” OR “Maritime pine” OR “ *Pinus pinea* ” OR “Stone pine” OR “ *Pinus sylvestris* ” OR “Scots pine” OR “ *Pinus uncinata* ” OR “Mountain pine” OR “ *Platanus orientalis* ” OR “Oriental plane” OR “ *Populus alba* ” OR “silver‐leaved poplar” OR “white poplar” OR “abell” OR “silver poplar” OR “silverleaf poplar” OR “silver‐leaf poplar” OR “silver leaved poplar” OR “ *Populus nigra* ” OR “black poplar” OR “ *Populus tremula* ” OR “European aspen” OR “trembling poplar” OR “ *Prunus avium* ” OR “sweet cherry” OR “bird cherry” OR “wild cherry” OR “ *Prunus cerasifera* ” OR “Cherry plum” OR “ *Prunus padus* ” OR “Bird cherry” OR “ *Prunus spinosa* ” OR “buckthorn” OR “ *Pyrus pyraster* ” OR “wild pear” OR “ *Quercus cerris* ” OR “Turkey oak” OR “Austrian oak” OR “Quercus coccifera” OR “Kermes oak” OR “ *Quercus frainetto* ” OR “Hungarian oak” OR “Italian oak” OR “ *Quercus ilex* ” OR “Holm oak” OR “Quercus pedunculiflora” OR “Grayish oak” OR “ *Quercus petraea* ” OR “durmast oak” OR “sessile oak” OR “cornish oak” OR “Quercus pubescens” OR “downy oak” OR “pubescent oak” OR “ *Quercus robur* ” OR “common oak” OR “english oak” OR “pedunculate oak” OR “ *Quercus suber* ” OR “Cork oak” OR “Quercus trojana” OR “Macedonian oak” OR “Quercus virgiliana” OR “Oak of Virgil” OR “ *Salix alba* ” OR “White willow” OR “ *Salix caprea* ” OR “Goat willow” OR “ *Sorbus aria* ” OR “whitebear” OR “beam tree” OR “chess apple” OR “ *Sorbus aucuparia* ” OR “rowan” OR “mountain ash” OR “ *Sorbus domestica* ” OR “service tree” OR “sorb tree” OR “whitty pear” OR “ *Sorbus intermedia* ” OR “Swedish whitebear” OR “ *Sorbus torminalis* ” OR “checker tree” OR “chequer tree” OR “wild service tree” OR “ *Taxus baccata* ” OR “yew” OR “ *Tilia cordata* ” OR ((“littleleaf” OR small*leaf OR small*leaved) AND (“lime” OR “linden”)) OR “ *Tilia platyphyllos* ” OR ((broad*leaved OR broad*leaf OR large*leaved OR large*leaf) AND (lime OR linden)) OR “ *Tilia tomentosa* ” OR “Silver lime” OR “Silver linden” OR “ *Ulmus carpinifolia* ” OR “ *Ulmus minor* ” OR “field elm” OR “small‐leaved elm” OR “ *Ulmus glabra* ” OR “Scots elm” OR “wych elm” OR “Ulmus laevis” OR “white elm” OR “fluttering elm” OR “Russian elm” OR “spreading elm”) AND (“local adaptation” OR “genetic heterogeneity” OR “intraspecific variation” OR genecolog* OR “population genomics” OR (provenance AND clima* AND respon*)))) OR AK = ((“ *Abies alba* ” OR “silver fir” OR “Abies borisii‐regis” OR “King Boris fir” OR “Abies bornmuelleriana” OR “Bornmuller's fir” OR “Bornmüller's fir” OR “Abies cephalonica” OR “Grecian fir” OR “Greek fir” OR “Abies nebrodensis” OR “Sicilian fir” OR “Abies pinsapo” OR “Spanish fir” OR “ *Acer campestre* ” OR “field maple” OR “hedge maple” OR “maser” OR “Acer monspessulanum” OR “French maple” OR “Montpellier maple” OR “ *Acer opalus* ” OR “Italian maple” OR “opalus maple” OR “ *Acer platanoides* ” OR “Norway maple” OR “ *Acer pseudoplatanus* ” OR “Sycamore” OR “ *Aesculus hippocastanum* ” OR “horse chestnut” OR “ *Alnus cordata* ” OR “Italian alder” OR “ *Alnus glutinosa* ” OR “black alder” OR “ *Alnus incana* ” OR “gray alder” OR “gray alder” OR “speckled alder” OR “ *Alnus viridis* ” OR “Green alder” OR “ *Betula pendula* ” OR “weeping birch” OR “warty birch” OR “ *Betula pubescens* ” OR “downy birch” OR “mountain birch” OR “silver birch” OR “swamp birch” OR “ *Carpinus betulus* ” OR “hornbeam” OR “ *Castanea sativa* ” OR “sweet chestnut” OR “Spanish chestnut” OR “ *Corylus avellana* ” OR “common hazel” OR “ *Corylus colurna* ” OR “Turkish hazel” OR “ *Celtis australis* ” OR “European nettle tree” OR “ *Cornus mas* ” OR “Cornelian cherry” OR “ *Cornus sanguinea* ” OR “Common dogwood” OR “ *Cupressus sempervirens* ” OR “Italian cypress” OR “Fagus orientalis” OR “Oriental beech” OR “ *Fagus sylvatica* ” OR “European beech” OR “Fagus taurica” OR “Crimean beech” OR “ *Frangula alnus* ” OR “Glossy buckthorn” OR “Fraxinus angustifolia” OR “narrow‐leafed ash” OR “ *Fraxinus excelsior* ” OR “european ash” OR “common ash” OR “ *Fraxinus ornus* ” OR “manna ash” OR “ *Ilex aquifolium* ” OR “Common holly” OR “ *Juglans regia* ” OR “walnut” OR “ *Juniperus communis* ” OR “Common juniper” OR “ *Juniperus excelsa* ” OR “Greek juniper” OR “ *Juniperus oxycedrus* ” OR “Prickly juniper” OR “ *Larix decidua* ” OR “European larch” OR “ *Malus sylvestris* ” OR “Wild apple” OR “Ostrya carpinifolia” OR “hop‐hornbeam” OR “hophornbeam” OR “ *Picea abies* ” OR “Norway spruce” OR “ *Picea omorika* ” OR “Serbian spruce” OR “ *Pinus brutia* ” OR “Brutia pine” OR “ *Pinus cembra* ” OR “Swiss stone pine” OR “ *Pinus halepensis* ” OR “Aleppo pine” OR “ *Pinus heldreichii* ” OR “Bosnian pine” OR “ *Pinus mugo* ” OR “Mountain pine” OR “ *Pinus nigra* ” OR “European black pine” OR “black pine” OR “ *Pinus peuce* ” OR “Macedonian pine” OR “ *Pinus pinaster* ” OR “Maritime pine” OR “ *Pinus pinea* ” OR “Stone pine” OR “ *Pinus sylvestris* ” OR “Scots pine” OR “ *Pinus uncinata* ” OR “Mountain pine” OR “ *Platanus orientalis* ” OR “Oriental plane” OR “ *Populus alba* ” OR “silver‐leaved poplar” OR “white poplar” OR “abell” OR “silver poplar” OR “silverleaf poplar” OR “silver‐leaf poplar” OR “silver leaved poplar” OR “ *Populus nigra* ” OR “black poplar” OR “ *Populus tremula* ” OR “European aspen” OR “trembling poplar” OR “ *Prunus avium* ” OR “sweet cherry” OR “bird cherry” OR “wild cherry” OR “ *Prunus cerasifera* ” OR “Cherry plum” OR “ *Prunus padus* ” OR “Bird cherry” OR “ *Prunus spinosa* ” OR “buckthorn” OR “ *Pyrus pyraster* ” OR “wild pear” OR “ *Quercus cerris* ” OR “Turkey oak” OR “Austrian oak” OR “Quercus coccifera” OR “Kermes oak” OR “ *Quercus frainetto* ” OR “Hungarian oak” OR “Italian oak” OR “ *Quercus ilex* ” OR “Holm oak” OR “Quercus pedunculiflora” OR “Grayish oak” OR “ *Quercus petraea* ” OR “durmast oak” OR “sessile oak” OR “cornish oak” OR “Quercus pubescens” OR “downy oak” OR “pubescent oak” OR “ *Quercus robur* ” OR “common oak” OR “english oak” OR “pedunculate oak” OR “ *Quercus suber* ” OR “Cork oak” OR “Quercus trojana” OR “Macedonian oak” OR “Quercus virgiliana” OR “Oak of Virgil” OR “ *Salix alba* ” OR “White willow” OR “ *Salix caprea* ” OR “Goat willow” OR “ *Sorbus aria* ” OR “whitebear” OR “beam tree” OR “chess apple” OR “ *Sorbus aucuparia* ” OR “rowan” OR “mountain ash” OR “ *Sorbus domestica* ” OR “service tree” OR “sorb tree” OR “whitty pear” OR “ *Sorbus intermedia* ” OR “Swedish whitebear” OR “ *Sorbus torminalis* ” OR “checker tree” OR “chequer tree” OR “wild service tree” OR “ *Taxus baccata* ” OR “yew” OR “ *Tilia cordata* ” OR ((“littleleaf” OR small*leaf OR small*leaved) AND (“lime” OR “linden”)) OR “ *Tilia platyphyllos* ” OR ((broad*leaved OR broad*leaf OR large*leaved OR large*leaf) AND (lime OR linden)) OR “ *Tilia tomentosa* ” OR “Silver lime” OR “Silver linden” OR “ *Ulmus carpinifolia* ” OR “ *Ulmus minor* ” OR “field elm” OR “small‐leaved elm” OR “ *Ulmus glabra* ” OR “Scots elm” OR “wych elm” OR “Ulmus laevis” OR “white elm” OR “fluttering elm” OR “Russian elm” OR “spreading elm”) AND (“local adaptation” OR “genetic heterogeneity” OR “intraspecific variation” OR genecolog* OR “population genomics” OR (provenance AND clima* AND respon*)))) OR KP = ((“ *Abies alba* ” OR “silver fir” OR “Abies borisii‐regis” OR “King Boris fir” OR “Abies bornmuelleriana” OR “Bornmuller's fir” OR “Bornmüller's fir” OR “Abies cephalonica” OR “Grecian fir” OR “Greek fir” OR “Abies nebrodensis” OR “Sicilian fir” OR “Abies pinsapo” OR “Spanish fir” OR “ *Acer campestre* ” OR “field maple” OR “hedge maple” OR “maser” OR “Acer monspessulanum” OR “French maple” OR “Montpellier maple” OR “ *Acer opalus* ” OR “Italian maple” OR “opalus maple” OR “ *Acer platanoides* ” OR “Norway maple” OR “ *Acer pseudoplatanus* ” OR “Sycamore” OR “ *Aesculus hippocastanum* ” OR “horse chestnut” OR “ *Alnus cordata* ” OR “Italian alder” OR “ *Alnus glutinosa* ” OR “black alder” OR “ *Alnus incana* ” OR “gray alder” OR “gray alder” OR “speckled alder” OR “ *Alnus viridis* ” OR “Green alder” OR “ *Betula pendula* ” OR “weeping birch” OR “warty birch” OR “ *Betula pubescens* ” OR “downy birch” OR “mountain birch” OR “silver birch” OR “swamp birch” OR “ *Carpinus betulus* ” OR “hornbeam” OR “ *Castanea sativa* ” OR “sweet chestnut” OR “Spanish chestnut” OR “ *Corylus avellana* ” OR “common hazel” OR “ *Corylus colurna* ” OR “Turkish hazel” OR “ *Celtis australis* ” OR “European nettle tree” OR “ *Cornus mas* ” OR “Cornelian cherry” OR “ *Cornus sanguinea* ” OR “Common dogwood” OR “ *Cupressus sempervirens* ” OR “Italian cypress” OR “Fagus orientalis” OR “Oriental beech” OR “ *Fagus sylvatica* ” OR “European beech” OR “Fagus taurica” OR “Crimean beech” OR “ *Frangula alnus* ” OR “Glossy buckthorn” OR “Fraxinus angustifolia” OR “narrow‐leafed ash” OR “ *Fraxinus excelsior* ” OR “european ash” OR “common ash” OR “ *Fraxinus ornus* ” OR “manna ash” OR “ *Ilex aquifolium* ” OR “Common holly” OR “ *Juglans regia* ” OR “walnut” OR “ *Juniperus communis* ” OR “Common juniper” OR “ *Juniperus excelsa* ” OR “Greek juniper” OR “ *Juniperus oxycedrus* ” OR “Prickly juniper” OR “ *Larix decidua* ” OR “European larch” OR “ *Malus sylvestris* ” OR “Wild apple” OR “Ostrya carpinifolia” OR “hop‐hornbeam” OR “hophornbeam” OR “ *Picea abies* ” OR “Norway spruce” OR “ *Picea omorika* ” OR “Serbian spruce” OR “ *Pinus brutia* ” OR “Brutia pine” OR “ *Pinus cembra* ” OR “Swiss stone pine” OR “ *Pinus halepensis* ” OR “Aleppo pine” OR “ *Pinus heldreichii* ” OR “Bosnian pine” OR “ *Pinus mugo* ” OR “Mountain pine” OR “ *Pinus nigra* ” OR “European black pine” OR “black pine” OR “ *Pinus peuce* ” OR “Macedonian pine” OR “ *Pinus pinaster* ” OR “Maritime pine” OR “ *Pinus pinea* ” OR “Stone pine” OR “ *Pinus sylvestris* ” OR “Scots pine” OR “ *Pinus uncinata* ” OR “Mountain pine” OR “ *Platanus orientalis* ” OR “Oriental plane” OR “ *Populus alba* ” OR “silver‐leaved poplar” OR “white poplar” OR “abell” OR “silver poplar” OR “silverleaf poplar” OR “silver‐leaf poplar” OR “silver leaved poplar” OR “ *Populus nigra* ” OR “black poplar” OR “ *Populus tremula* ” OR “European aspen” OR “trembling poplar” OR “ *Prunus avium* ” OR “sweet cherry” OR “bird cherry” OR “wild cherry” OR “ *Prunus cerasifera* ” OR “Cherry plum” OR “ *Prunus padus* ” OR “Bird cherry” OR “ *Prunus spinosa* ” OR “buckthorn” OR “ *Pyrus pyraster* ” OR “wild pear” OR “ *Quercus cerris* ” OR “Turkey oak” OR “Austrian oak” OR “Quercus coccifera” OR “Kermes oak” OR “ *Quercus frainetto* ” OR “Hungarian oak” OR “Italian oak” OR “ *Quercus ilex* ” OR “Holm oak” OR “Quercus pedunculiflora” OR “Grayish oak” OR “ *Quercus petraea* ” OR “durmast oak” OR “sessile oak” OR “cornish oak” OR “Quercus pubescens” OR “downy oak” OR “pubescent oak” OR “ *Quercus robur* ” OR “common oak” OR “english oak” OR “pedunculate oak” OR “ *Quercus suber* ” OR “Cork oak” OR “Quercus trojana” OR “Macedonian oak” OR “Quercus virgiliana” OR “Oak of Virgil” OR “ *Salix alba* ” OR “White willow” OR “ *Salix caprea* ” OR “Goat willow” OR “ *Sorbus aria* ” OR “whitebear” OR “beam tree” OR “chess apple” OR “ *Sorbus aucuparia* ” OR “rowan” OR “mountain ash” OR “ *Sorbus domestica* ” OR “service tree” OR “sorb tree” OR “whitty pear” OR “ *Sorbus intermedia* ” OR “Swedish whitebear” OR “ *Sorbus torminalis* ” OR “checker tree” OR “chequer tree” OR “wild service tree” OR “ *Taxus baccata* ” OR “yew” OR “ *Tilia cordata* ” OR ((“littleleaf” OR small*leaf OR small*leaved) AND (“lime” OR “linden”)) OR “ *Tilia platyphyllos* ” OR ((broad*leaved OR broad*leaf OR large*leaved OR large*leaf) AND (lime OR linden)) OR “ *Tilia tomentosa* ” OR “Silver lime” OR “Silver linden” OR “ *Ulmus carpinifolia* ” OR “ *Ulmus minor* ” OR “field elm” OR “small‐leaved elm” OR “ *Ulmus glabra* ” OR “Scots elm” OR “wych elm” OR “Ulmus laevis” OR “white elm” OR “fluttering elm” OR “Russian elm” OR “spreading elm”) AND (“local adaptation” OR “genetic heterogeneity” OR “intraspecific variation” OR genecolog* OR “population genomics” OR (provenance AND clima* AND respon*)))

#### Scopus (February 02, 2022)

TITLE‐ABS‐KEY((“ *Abies alba* ” OR “silver fir” OR “Abies borisii‐regis” OR “King Boris fir” OR “Abies bornmuelleriana” OR “Bornmuller's fir” OR “Bornmüller's fir” OR “Abies cephalonica” OR “Grecian fir” OR “Greek fir” OR “Abies nebrodensis” OR “Sicilian fir” OR “Abies pinsapo” OR “Spanish fir” OR “ *Acer campestre* ” OR “field maple” OR “hedge maple” OR “maser” OR “Acer monspessulanum” OR “French maple” OR “Montpellier maple” OR “ *Acer opalus* ” OR “Italian maple” OR “opalus maple” OR “ *Acer platanoides* ” OR “Norway maple” OR “ *Acer pseudoplatanus* ” OR “Sycamore” OR “ *Aesculus hippocastanum* ” OR “horse chestnut” OR “ *Alnus cordata* ” OR “Italian alder” OR “ *Alnus glutinosa* ” OR “black alder” OR “ *Alnus incana* ” OR “gray alder” OR “gray alder” OR “speckled alder” OR “ *Alnus viridis* ” OR “Green alder” OR “ *Betula pendula* ” OR “weeping birch” OR “warty birch” OR “ *Betula pubescens* ” OR “downy birch” OR “mountain birch” OR “silver birch” OR “swamp birch” OR “ *Carpinus betulus* ” OR “hornbeam” OR “ *Castanea sativa* ” OR “sweet chestnut” OR “Spanish chestnut” OR “ *Corylus avellana* ” OR “common hazel” OR “ *Corylus colurna* ” OR “Turkish hazel” OR “ *Celtis australis* ” OR “European nettle tree” OR “ *Cornus mas* ” OR “Cornelian cherry” OR “ *Cornus sanguinea* ” OR “Common dogwood” OR “ *Cupressus sempervirens* ” OR “Italian cypress” OR “Fagus orientalis” OR “Oriental beech” OR “ *Fagus sylvatica* ” OR “European beech” OR “Fagus taurica” OR “Crimean beech” OR “ *Frangula alnus* ” OR “Glossy buckthorn” OR “Fraxinus angustifolia” OR “narrow‐leafed ash” OR “ *Fraxinus excelsior* ” OR “european ash” OR “common ash” OR “ *Fraxinus ornus* ” OR “manna ash” OR “ *Ilex aquifolium* ” OR “Common holly” OR “ *Juglans regia* ” OR “walnut” OR “ *Juniperus communis* ” OR “Common juniper” OR “ *Juniperus excelsa* ” OR “Greek juniper” OR “ *Juniperus oxycedrus* ” OR “Prickly juniper” OR “ *Larix decidua* ” OR “European larch” OR “ *Malus sylvestris* ” OR “Wild apple” OR “Ostrya carpinifolia” OR “hop‐hornbeam” OR “hophornbeam” OR “ *Picea abies* ” OR “Norway spruce” OR “ *Picea omorika* ” OR “Serbian spruce” OR “ *Pinus brutia* ” OR “Brutia pine” OR “ *Pinus cembra* ” OR “Swiss stone pine” OR “ *Pinus halepensis* ” OR “Aleppo pine” OR “ *Pinus heldreichii* ” OR “Bosnian pine” OR “ *Pinus mugo* ” OR “Mountain pine” OR “ *Pinus nigra* ” OR “European black pine” OR “black pine” OR “ *Pinus peuce* ” OR “Macedonian pine” OR “ *Pinus pinaster* ” OR “Maritime pine” OR “ *Pinus pinea* ” OR “Stone pine” OR “ *Pinus sylvestris* ” OR “Scots pine” OR “ *Pinus uncinata* ” OR “Mountain pine” OR “ *Platanus orientalis* ” OR “Oriental plane” OR “ *Populus alba* ” OR “silver‐leaved poplar” OR “white poplar” OR “abele” OR “silver poplar” OR “silverleaf poplar” OR “silver‐leaf poplar” OR “silver leaved poplar” OR “ *Populus nigra* ” OR “black poplar” OR “ *Populus tremula* ” OR “European aspen” OR “trembling poplar” OR “ *Prunus avium* ” OR “sweet cherry” OR “bird cherry” OR “wild cherry” OR “ *Prunus cerasifera* ” OR “Cherry plum” OR “ *Prunus padus* ” OR “Bird cherry” OR “ *Prunus spinosa* ” OR “Blackthorn” OR “ *Pyrus pyraster* ” OR “wild pear” OR “ *Quercus cerris* ” OR “Turkey oak” OR “Austrian oak” OR “Quercus coccifera” OR “Kermes oak” OR “ *Quercus frainetto* ” OR “Hungarian oak” OR “Italian oak” OR “ *Quercus ilex* ” OR “Holm oak” OR “Quercus pedunculiflora” OR “Grayish oak” OR “ *Quercus petraea* ” OR “durmast oak” OR “sessile oak” OR “cornish oak” OR “Quercus pubescens” OR “downy oak” OR “pubescent oak” OR “ *Quercus robur* ” OR “common oak” OR “english oak” OR “pedunculate oak” OR “ *Quercus suber* ” OR “Cork oak” OR “Quercus trojana” OR “Macedonian oak” OR “Quercus virgiliana” OR “Oak of Virgil” OR “ *Salix alba* ” OR “White willow” OR “ *Salix caprea* ” OR “Goat willow” OR “ *Sorbus aria* ” OR “whitebeam” OR “beam tree” OR “chess apple” OR “ *Sorbus aucuparia* ” OR “rowan” OR “mountain ash” OR “ *Sorbus domestica* ” OR “service tree” OR “sorb tree” OR “whitty pear” OR “ *Sorbus intermedia* ” OR “Swedish whitebeam” OR “ *Sorbus torminalis* ” OR “checker tree” OR “chequer tree” OR “wild service tree” OR “ *Taxus baccata* ” OR “yew” OR “ *Tilia cordata* ” OR ((“littleleaf” OR small*leaf OR small*leaved) AND (“lime” OR “linden”)) OR “ *Tilia platyphyllos* ” OR ((broad*leaved OR broad*leaf OR large*leaved OR large*leaf) AND (lime OR linden)) OR “ *Tilia tomentosa* ” OR “Silver lime” OR “Silver linden” OR “ *Ulmus carpinifolia* ” OR “ *Ulmus minor* ” OR “field elm” OR “small‐leaved elm” OR “ *Ulmus glabra* ” OR “Scots elm” OR “wych elm” OR “Ulmus laevis” OR “white elm” OR “fluttering elm” OR “Russian elm” OR “spreading elm”) AND (“local adaptation” OR “genetic heterogeneity” OR “intraspecific variation” OR genecolog* OR “population genomics” OR (provenance AND clima* AND respon*))).

### Selection Criteria List

- Natural European tree species (exclude introduced species, cultivars, breeds, hybrids).
- Studies that treat intraspecific variation, local adaptation, genetic heterogeneity, population genomics (among population differences), provenance climate responses, genecological studies or similar.
- Only studies in English.
- Exclude studies that relate intraspecific variation to management or competition or mixture but not to genetic variation or climate.
- Include studies that do not relate local adaptation to climate but generally to intraspecific variation between populations (e.g., also range groups).
- Exclude ecological studies.
- Exclude Reviews.
- Exclude palaeoecological studies.
- Comparison of at least two populations/regions/localities, etc.
- Exclude studies that do not compare to populations via translocation or experimental treatments (i.e., studies that do not correct for differences in local climate).
- Only functional traits excluding chemical traits and studies that may not be different in locations or hard to find (e.g., pollution/wood quality, material science).
- Exclude studies that are purely genetic and do not include functional traits.

**TABLE A1:** List of extracted data.

Data type	Description	Notes
Year	Year of publication	
Species	Name of species studied	
Number of Species	Number of different species studied in publication	The total number examined. Only native Europeans extracted. If nonnatives were also included in article they were still counted in the number of species
Provenances	Number of provenances (i.e., single populations) studied	If different per trial → median; hybrids → single provenance (article not included if only hybrids); if populations and provenances given → populations (i.e., smaller unit)
Locations/treatments	Number of trials or treatments	For translocation or manipulation experiment, respectively
Type of analysis	Type of analysis identified	ANOVA, regression (linear), regression (quadratic), correlation, nonparametric
Type of study	What overall type of study was conducted (e.g., Common garden, growth chamber, full translocation)	Common Garden: actual planting; Greenhouse: in greenhouses/pots; growth chamber: in completely artificial growth chamber
Age	What was the age of the examined individuals	Several years sampled → separated by comma; if different ages between provenances sampled → separated by semicolon; if age of planting given as range → youngest age taken
Age group	Age assigned to one of three groups	1–5 years: seedling, 5–20: juvenile tree, > 20: mature tree
Assessment	Short written summary of data analysis	Read through methods and result and only extract information that is relevant for local adaptation (e.g., in consideration of Table 1)
P countries	Countries where provenances were taken	Every country counted once per case (two‐digit ISO code), no matter the number of provenances
T countries	Countries where trials were conducted	Every country counted once per case (two‐digit ISO code), no matter the number of trials
P part of range	Part of range where provenances where taken (i.e., central, northern, southern, northern margin, southern margin) according to EUFORGEN distribution map or otherwise mentioned in the article	Across natural range = central, southern, northern; margin if explicitly mentioned or visible from map
T part of range	Part of range where trials where conducted (i.e., central, northern, southern, northern margin, southern margin) according to see above	Across natural range = central, southern, northern; margin if explicitly mentioned or visible from map
Reason for exclusion	In case of exclusion statement of reason	
Trait	Trait that was studied	
Effect	The effect studied, that is, provenance–effect, provenance–environment interaction or climate variables as predictors	TD added to predictor if transfer distance and not climate at origin was used
Significance	The significance of the effect as Yes/No/Not available	0 if predictor had no sign. effect, 1 if it had sign. effect, NA if predictor was not studied

## Appendix B Complete List of Traits

**Allocation**—Taits that describe the distribution of resources within a plant (e.g., ratios of biomass):

Apical dominance ratio

Below/aboveground biomass ratio

Branch area sum to stem cross‐sectional area ratio

Bud bank characteristics

Coarse root fraction

Coarse root fraction increment

Fine root fraction

Fine root fraction increment

Fine root: leaf surface area ratio

Leaf area ratio

Leaf area to total biomass ratio

Leaf area/plant weight ratio

Leaf biomass fraction

Leaf biomass fraction (in drought experiment)

Leaf dry mass ratios (LWR)

Leaf fresh weight/dry weight ratio

Leaf mass ratio (in drought experiment)

Leaf weight/plant weight ratio

Needle mass to branch cross‐sectional area ratio

Needle mass to stem cross‐sectional area ratio

Needle/wood biomass ratio

Proportional biomass allocation to needles

Proportional biomass allocation to root

Proportional biomass allocation to stem

Ratio of terminal leader length to number of apical buds

Root biomass fraction

Root dry mass ratios (RWR)

Root tip to shoot ratio

Root to shoot ratio

Root/shoot biomass ratio

Root/shoot biomass ratio (dry conditions)

Root/shoot biomass ratio (in drought experiment)

Root/shoot biomass ratio (watered conditions)

Root/shoot length ratio

Root/shoot ratio

Root:shoot

Root:shoot length

Sapwood to leaf area ratio (Huber value)

Shoot dry mass ratios (ShWR)

Shoot/root biomass ratio

Shoot/root biomass ratio (in autumn fertilization experiment)

Stem biomass fraction

Stem dry mass ratios (StWR)

Stem mass ratio (in drought experiment)

**Drought tolerance:**—Direct reactions to frost (e.g., leaf damages, growth differential between drought treatment and control), traits that were only indirectly related or without comparison between treatment and control were put into other traitgroups (e.g., height in drought treatment was grouped to growth) assuming the treatment was only an envrionmental factor:

Absolute height growth drought response (Annual)

Absolute height growth drought response (Jan–Jun)

Absolute height increment drought response (Annual)

Absolute height increment drought response (Jan–Jun)

Absolute needle increment drought response (Jan–Jun)

Absolute needle increment drought response (Jul–Aug)

Critical temperature for PSII stability

Diameter growth drought response (Annual)

Diameter increment drought response (Annual)

Difference in shoot increment between wet and dry year

Drought induced mean diameter reduction

Drought induced mean height reduction

Drought induced mortality

Drought recovery

Drought relative resilience

Drought resilience

Drought resistance

Drought resistance (earlywood)

Drought resistance (latewood)

Drought sensitivity of diameter increment

Drought sensitivity of height increment

Height differential due to drought

Leaf damage (in drought experiment)

Leaf damage due to drought

Modulus of elasticity (in dry treatment relative to moist treatment)

Osmotic potential (recovery after drought experiment)

Photosynthetic rate (recovery after drought experiment)

Predawn water potential (recovery after drought experiment)

Proline content (recovery after drought experiment)

Relative electolyte leakage after incubation in paraquat (in drought experiment)

Relative electolyte leakage after incubation in water (in drought experiment)

Relative height growth drought response (Annual)

Relative height growth drought response (Jan–Jun)

Relative height increment drought response (Annual).

Relative height increment drought response (Jan‐Jun)

Relative needle increment drought response (Jan‐Jun)

Relative needle increment drought response (Jul‐Aug)

Relative needle water content (recovery after drought experiment)

Severity of heat stress (low value = high severity)

Stomatal conductance (recovery after drought experiment)

Timing of leaf wilting in drought t

Xylem vulnerability to cavitation

**Frost tolerance**—Direct reactions to frost (e.g., leaf damages, growth differential between frost treatment and control), traits that were only indirectly related or without comparison between treatment and control were put into other traitgroups (e.g., height in frost treatment was grouped to growth) assuming the treatment was only an envrionmental factor:

Bud frost damage in autumn

Bud frost damage in spring

Bud frost tolerance

Frost damage

Frost damage in autumn

Frost damage in spring

Height differential due to frost

Late frost damage

Leaf damage differential due to frost

Leaf damage due to frost

LT50 due to frost (in Maximum photochemical efficiency of PSII in cortical bark chlorenchyma)

LT50 due to frost (in Maximum photochemical efficiency of PSII in needles)

LT50 cold hardiness (relative electrolyte leakage method)

Needle frost damage in autumn

Needle frost damage in spring

Root frost tolerance

Stem frost damage in autumn

Stem frost damage in spring

Visual frost damage

Vitality

**Growth—**Amount or increment of organic material (e.g., height, diameter, biomass, thickness) irrespective of organ (roots, branches, stem, leaves):

Above ground biomass

Above ground biomass increment

Aboveground biomass

Absolute height growth rate

Adult height

Annual branch sapwood area increment

Annual growth

Annual shoot increment

Annual stem circumference increase

Apical biomass

Apical length

Average normalized DBH

Average root system length

BAI

Basal area increment

Belowground biomass

Biom

Branch number increment

Branch thickness

Bud biomass

Central shoot diameter

Central shoot diameter increment

Central shoot length

Central shoot length increment

Change in height per change in MAT (slope)

Coarse root fresh weight

Coarse root mass

Collar biomass

Corrected average height

Crown expansion

Crown expansion increment

DBH

DBH increment

Diameter

Diameter increment

Diameter selected trees

Dominant branches diameter

Dominance compared to neighbor

Estimated height at age 100

Fine root diameter

Fine root fresh weight

Fine root mass

Fine root surface area

Fine root surface as root forks

Fine root surface as root forks increment

Fine root surface as root tips

Fine root surface as root tips increment

Fresh weight

Growth response

Height

Height (logtransformed)

Height after clipping

Height at 15 years

Height derivate

Height growth rates

Height increment

Height

Hypocotyl height

Lateral biomass

Lateral length

Leaf biomass

Leaf dry mass

Leaf dry matter content

Leaf dry weight

Leaf fresh weight

Leaf fresh weight/dry weight ratio

Leaf number increment

Maximum growth rate

Maximum length of longest leaf increment

Mean annual increment of yield during rotation

Mean climate sensitivity of diameter increment

Mean climate sensitivity of height growth

Mean height

Mean radial increment

Mean ring width

Mean tree volume

Needle biomass

Needle increment

Needle mass

Proximal taproot biomass

Radial increment

Random effect of population on total biomass

Relative annual height growth

Relative diameter growth rate

Relative growth rate (weight)

Relative height growth rate

Relative height growth rate corrected for initial height

Residual height

Ring width

Root biomass

Root collar diameter

Root collar diameter

Root dry weight

Root fresh weight/dry weight ratio

Root growth potential

Root length

Root length increment

Root mass

Root‐specific surface area

Root‐collar diameter increment

Sapling length

Scaled tree height increment

Schoot diameter (after cutting back 1 year after drought t)

Seedling size (volume)

Shoot biomass

Shoot dry weight

Shoot emergence after drought t

Shoot height

Shoot length

Shoot mass

Specific root length

Specific stem length

Stand volume

Stem biomass

Stem diameter

Stem fresh weight

Stem length

Stem mass

Stem volume

Surface equally fine root length

Surface equally fine root length increment

Tap root length

Taproot length

Terminal shoot length

Total biomass

Total dry mass

Total dry mass increment

Total dry weight

Total plant mass

Total root length

Total root length increment

Tree ring width

Unit leaf rate

Volume

**Leaf anatomy/Leaf morphology**—Morphological structure of and anatomical structure inside leaves (e.g., shape, number of veins):

Abaxial epidermis thickness

Adaxial epidermis thickness

Density of trichomes

Distance between third and fourth vein

Lamina length

Lamina thickness

Lamina width

Leaf area

Leaf density

Leaf length

Leaf length/width ratio

Leaf mass area

Leaf mass per area

Leaf morphology

Leaf shape

Leaf size

Leaf thickness

Leaf vein density

Leaf width

Length of stomata guard cells

Main‐vein diameter

Major vein density

Maximum length of longest leaf

Mean leaf area

NDVI (leaf area)

Needle cross section (mm^2^)

Needle cuticula (mm^2^)

Needle cuticula (mycrometer)

Needle length

Needle mesophyll (%)

Needle mesophyll (mm^2^)

Needle phloem (%)

Needle phloem (mm^2^)

Needle resin duct (%)

Needle resin duct (mm^2^)

Needle resin duct number

Needle width

Needle xylem (%)

Needle xylem (mm^2^)

Number of pairs of secondary veins

Number of veins on the left side of the leaf

Number of veins on the right side of the leaf

OSAVI (leaf area)

Palisade parenchyma thickness

Palisade parenchyma/mesophyll ratio

Petiole length

Sclerenchyma thickness

Second leaf size

Specific leaf area

Spongy parenchyma thickness

Stomatal density

Stomatal shape (length/width)

Stomatal size (length*width)

Vein density

Whole plant leaf area

Width of stomata guard cells

**Leaf chem. comp.:** Chemical composition of leaves:

% leaf nitrogen content per leaf mass basis

(−)‐epicatechin

(+)‐catechin

3,4‐dihydroxydihydrofuranone

Abscisic acid content

Abscisic acid

Alpha‐tocopherol to chlorophyll molar ratio

Area based nitrogen content

ARI2 (anthocyanin)

Caffeic acid

Carbon to nitrogen ratio

Carbon–nitrogen ratio

Carotene

Carotenoid content

Carotenoids

Chlorogenic acid

Chlorophyll a

Chlorophyll a + b

Chlorophyll a content

Chlorophyll a/b

Chlorophyll a + b

Chlorophyll b

Chlorophyll b content

Chlorophyll/Carotenoid ratio

Chlorophylls a + b content

Citric acid

CRI2 (carotenoid)

D‐chiro‐inositol

Degree of deepoxidation (ratio between antheraxanthin plus zeaxanthin (A + Z) and xanthophyll cycle pigments (V + A + Z))

Dehydroascorbic acid

D‐fructose

D‐galactose

D‐glucose

Disaccharide

D‐levoglucosan

D‐mannitol

D‐myo‐inositol

D‐myo‐inositol phosphate

D‐ribose

Epicuticular waxes

Ferulic acid

Fumaric acid

Glucose

Glucose dehydro derivative

Glutamic acid

Glyceric acid

Glycerol

Glycerol‐3‐phosphate (G3P)

Glycine

Glycolic acid

Hydrophilic antioxidants

Indole acetic acid content

Jasmonic acid content

Kaempferol

L‐alanine

L‐aspartic acid

L‐cysteine

Leaf C

Leaf C content

Leaf C/N

Leaf Ca concentration

Leaf Cu concentration

Leaf Fe concentration

Leaf K concentration

Leaf Mg concentration

Leaf Mn concentration

Leaf N

Leaf N concentration

Leaf N content

Leaf nitrogen composition

Leaf nitrogen content per unit mass

Leaf nitrogen isotope signature

Leaf P concentration

Leaf soluble sugar concentration

Leaf starch concentration

Leaf Zn concentration

Linoleic acid

Lipophilic antioxidants

L‐isoleucine

L‐proline

L‐serine

L‐threonine

L‐valine

Malic acid

Maltose

Mass‐specific foliar calcium content

Mass‐specific foliar magnesium content

Mass‐specific foliar nitrogen content

Mass‐specific foliar phosphor content

Mass‐specific foliar potassium content

Needle %N concentration

Needle C (%)

Needle Ca (%)

Needle K (%)

Needle Mg (%)

Needle N

Needle N (%)

Needle P (%)

Neophytadiene

Nitrogen leaf content per unit mass

Oleic acid

Optical index for leaf adaxial epidermal flavonols

Optical index of hydroxy‐cinnamic acids

Palmitic acid

PC1 Nutrients (Ca + Mg + Zn)

PC1 Pigments (Chlorophyll a)

PC2 Nutrients (N + K + Na)

PC2 Pigments (Chlorophyll b)

PC3 Nutrients (Mn + Cu)

PC3 Pigments (Carotenoids)

P‐coumaric acid

Phosphate

Phytol

Proline

Proline concentration

Proline content

Pyroglutamic acid

Quercetin

Quinic acid

Salicylic acid content

Shikimic acid

Soluble protein concentration

Soluble sugars

Solute accumulation at 80% relative water content

Squalene

Starch

Stearic acid

Succinic acid

Sucrose

Sugar acid

TCARI* (chlorophyll concentration)

TCARI/OSAVI* (chlorophyll concentration)

Threonic acid

Total chlorophyll content

Total non‐structural carbohydrate concentrations

Total phenol concentration

Totoal chlorophyll leaf content per unit mass

α‐linolenic acid

α‐tocopherol

β‐sitosterol

Υ‐aminobutiric acid (GABA)

**Phenology**—Traits related to yearly reoccurring events in tree life cycles like bud development, leaf senescence but also vegetation period length (time between the former both):

Bud burst

Bud burst (Julian days)

Bud burst at base stem

Bud burst at top of shoot

Bud burst duration

Bud burst timing

Bud dormancy

Bud flush

Bud flush (daylength)

Bud flush (Julian days)

Bud flush (temperature sums)

Bud flush duration

Bud set

Bud swelling

Critical chilling requirements

Critical forcing requirements

Defoliation

Dry apex

Fifth‐year terminal bud break

Flower opening

Fourth‐year lateral bud break

Fourth‐year terminal bud break

Growing season length

Growth cessation (Julian days)

Growth duration

Growth initiation

Growth onset

Height growth cessation

Height growth duration

Height increment cessation (length of night at)

Inflection point of Gompertz function

Juvenile ontogeny

Lateral flushing

Leaf duration

Leaf fall

Leaf onset

Leaf senescence

Leaf senescence (Julian days)

Leaf senescence at base stem

Leaf senescence at top of shoot

Leaf senescence duration

Leaf unfolding

Length of second flushing

Needle foliation onset

Needle foliation time

Needle unfolding

Needles unfolded

Needles unfolding

Ontogenetic score

Polycyclism

Provenance‐specific temperature sum requirement for bud burst

Relative chlorophyll content (autumn)

Residual bud flush (temperature sums)

Residual growing season length

Residual growth cessation (Julian days)

Second flushing

Shoot elongation period duration

Shoot phenology

Terminal flushing

Time lag

Timing of bud set

Total degree days at 50% bud set

Velocity of bud break (Julian days)

Velocity of bud break (temperature sums)

**Physiology—**General biochemical and biophysical processes within trees (e.g., photosynthesis, conductance):

Actual PSII efficiency

Area below rapid light curve

Assimilation rate

Basal fluorescence of chlorophyll a

Bulk modulus of tissue elasticity near full hydration (summer cell wall elasticity, elastic adjustment)

Carbon isotope composition

Carbon isotope composition of wood alpha‐cellulose (intrinsic water‐use efficiency)

Carbon isotope discrimination

Carbon isotopic fractioning

Cell volume

Chlorophyll fluorescence (RLCs)

Critical temperature reducing 15% of maximal photochemical efficiency

Critical temperature reducing 50% of maximal photochemical efficiency

Cumulated water loss

Cuticular transpiration

Daily difference of leaf water potential

Dark respiration

Density of active PSII reaction centers per chlorophyll

Dissipation of energy excess

Diurnal transpiration rate

Effective fluorescence yield

Effective quantum yield of photosystem II

Elastic adjustment (early‐late)

Elastic adjustment (moist‐dry)

Electron transport rate

Empirical leaf‐specific conductivity

Empirical specific conductivity

Fine root carbon isotope signature

Green‐to‐red reflectance

Green‐to‐yellow reflectance

Groundwater water usage

Hydraulic conductivity

Hydrogen isotopic composition of xylem water

Instantaneous water use efficiency

Intercellular CO2 concentration

Internal CO2

Intrinsic water use efficiency

Intrinsic water‐use efficiency

Isotopic composition of xylem water (water uptake, H)

Isotopic composition of xylem water (water uptake, O)

Leaf carbon isotope signature

Leaf reflectance

Leaf relative water content

Leaf specific conductivity

Leaf specific hydraulic conductivity

Leaf water content

Leaf water potential

Leaf water potential (predawn and relative: Predawn/Midday)

Leaf water potential at full hydration

Leaf water potential at turgor loss point

Light‐saturated net photosynthesis

Light‐saturated net photosynthesis:respiration rates

Loss of hydraulic conductivity

Lower soil water usage

Mass‐based maximum photosynthetic rate

Maximal photochemical efficiency

Maximum assimilation rate

Maximum bulk modulus of elasticity

Maximum carboxylation rate

Maximum electron transport rate

Maximum fluorescence yield

Maximum photochemical efficiency of PSII

Maximum photosynthetic electron flux

Maximum quantum yield of photosystem II

Mean chlorphyll fluorescence

Mean intrinsic water‐use efficiency

Mean needle light‐saturated net photosynthesis

Mean of net assimilation rate in situ

Mean predawn leaf water potential

Mean relative water content

Mean stomatal conductance

Mesophyll CO2 conductance

Mesophyll conductance

Midday leaf water potential

Modulus of elasticity

Needle carbon isotope signature

Net assimilation rate under light‐saturated conditions

Net photosynthesis per leaf area

Net photosynthesis per leaf mass

Net photosynthetic rate

Nitrogen isotopic fractioning

Nocturnal transpiration rate

Non‐photochemical quenching

Number of active reaction centers per antenna

Oleoresin flow

Osmotic adjustment

Osmotic adjustment (early‐late)

Osmotic adjustment (moist‐dry)

Osmotic potential at full turgor

Osmotic potential at turgor loss point

Oxygen isotopic composition of xylem water

PC1 of chlorophyll fluorescence (maximum quantum yield and others)

PC2 of chlorophyll fluorescence (variables expressed per unit of cross section)

PC3 of chlorophyll fluorescence (variables expressed per unit of reaction center)

Performance index on absorption basis

Performance index on absorption basis (after drought)

Performance of xanthophyllcycle (xantophyll cycle pigments (V + A + Z)/chlorophyll)

Photochemical quenching

Photosynthesis

Photosynthetic nitrogen use efficiency

Photosynthetic nitrogen‐use efficiency

Photosynthetic performance index

Photosynthetic rate

Potential conductance index

Predawn leaf water potential

Predawn needle water potential

Predawn twig water potential

Predawn water potential

Proportion of unproductive to productive water lass

Quantum yield (recovery)

Quantum yield (spring)

Quantum yield (summer)

Quantum yield of photosystem II

Ratio of internal to ambient CO2

Reduction of maximum efficiency of PSII due to flooding

Relative needle water content

Relative nitrogen allocation to bioenergetics

Relative nitrogen allocation to caroxylation

Relative nitrogen allocation to light harvesting complexed

Relative symplastic water content at turgor loss point

Relative water content

Relative water content at point of stomatal closure

Relative water content at point of stomatal closure (in summer)

Relative water content at point of stomatal closure (in winter)

Relative water content at predawn

Relative water content at turgor loss point

Relative−/total−/a−/b‐ chlorophyll content

Respiration rates

Slope of the xylem embolism vulnerability curve

Soil‐leaf hydraulic conductance

Specific hydraulic conductivity

Stem specific hydraulic conductance

Stem specific hydraulic conductivity

Stem water potential

Stomatal conductance

Stomatal conductance

Stomatal conductance at noon

Stomatal water vapor conductance under light‐saturated conditions

Temperature at which the value of variable fluorescence at the K‐step started to rapidly increase (high value = high damage)

Temperature threshold of PSII resistance

Theoretical leaf‐specific conductivity

Theoretical specific conductivity

Transpiration

Transpiration efficiency (total drymass—initial mean total drymass)

Transpiration rate

Turgor potential at full hydration

Upper soil water usage

Water content at the turgor loss point

Water use efficiency

WBI (leaf water content)

Xylem Pressure at 12% loss of hydraulic conductance

Xylem Pressure at 50% loss of hydraulic conductance

Xylem Pressure at 88% loss of hydraulic conductance

Xylem pressure potential

**Plant—animal interactions**—Interactions between the trees with animals (e.g., predation, infestation):

Bud consumption by pest

Duration of wilting symptoms due to nematodes

End of wilting symptoms due to nematodes

Leaf defoliation due to processionary moth

Leaf pest damage

Mortality due to nematode

Start of wilting symptoms due to nematodes

Wilting symptom development

**Plant morphology**—Morphological appearance of the complete tree (e.g., stem straightness):

Apical dominance

Average growth form of lateral shoots

Average growth form of proleptic shoots on terminal shoot

Branch angle

Branch number

Branches finesse

Branch sapwood area

Branchiness

Branching intensity

Buds on leader shoot

Crown diameter

Crown form

Crown slenderness

Crown width

Growth form of additional flushes

Growth form of first flush

Growth form of the lateral shoot

Growth form of the terminal shoot

Knots

Leaf number

Leaning

Length of longest branch

Lost top

Multistemming

Multistemming

Number apical buds

Number basal buds

Number collar buds

Number medial buds

Number of branches

Number of branches per whorl

Number of buds

Number of dwarf shoots

Number of lateral stems

Number of leaves

Number of leaves per shoot

Number of meristems along terminal shoots

Number of root tips

Number of secondary branches

Number of shoots

Number of stems

Number of whorls

Occurrence of proleptic lateral buds

Presence of lateral branches

Reaction location

Reaction place to clipping

Reaction to clipping

Reaction type

Relative stem taper

Second flush type

Shoot number

Slenderness (in autumn fertilization experiment)

Slenderness ratio

Specific stem length

Spike knots

Stem and crown form

Stem defects

Stem form

Stem quality

Stem straightness

Straightness

Terminal shoot growth form

Total leaf area

Total leaf area per shoot

Whorl shoots

**Plasticity indices**—Trait variation as reaction to different environmental conditions:

Coefficient of variation of phenotypic response

Phenotypic plasticity index

Plasticity index

Root/shoot ratio plasticity

Taproot length plasticity

**Reproduction—**Traits related to reproductive success like fruit onset, germination success:

Cumulative fitness

Cumulative germination percentage

Developmental time

Emergence

Emergence probability

Emergence rate

Emergence time

Empathy seeds

Empathy seeds %

Female median threshold size for first reproduction

Female reproductive biomass

Final germination percentage

Germination

Germination capacity (%)

Germination percentage

Germination rate

Germination speed

Male median threshold size for first reproduction

Male reproductive biomass

Mean emergence rate

Mean germination time

Mean germination value

Nonemergence probability

Number of seedlings

Random effect of population on seed mass

Relative cumulative percentage germination

Reproduction probability

Reproductive allocation

Seed mass

Time to 50% germination

**Root chem. comp**.—Chemical composition of roots:

Fine root C

Fine root N

Root C/N

Root total C

Root total N

**Survival—**Number of surviving individuals, also including mortality—number of eliminated individuals:

Annual survival

Annual survival (in dry year)

Change in survival per change in MAT (slope)

Cumulative survival

Long‐term survival

Mean survival rate

Mortality

Survival

Survival probability

Survival rate

Survival time

Total fitness (height × survival)

Winter survival

**Stem chem. comp.—**Chemical composition of stems:

Soluble sugar concentration in stem

Starch concentration in stem

Starch concentration in stem

**Wood anatomy—**Anatomical structure of wood (e.g., density):

95% quantile of vessel area

Cumulative vessel area

CWT (earlywood)

CWT (latewood)

Early/latewood width ratio

Earlywood density

Earlywood response to summer temperature

Earlywood width

Hydraulically weighted vessel diameter

Interannual density fluctuation (type E)

Interannual density fluctuation (type L)

Interannual density fluctuation (Type L+).

Latewood density

Latewood percent

Latewood proportion

Latewood width

LCA (earlywood)

LCA (latewood)

Long‐term earlywood

Lumen to sapwood area ratio

Maximum wood density

Mean ring density

Mean vessel area

Minimum wood density

Potential conductive area

Short term earlywood

Transition wood width

Vessel area

Vessel area fraction

Vessel density

Vessel diameter

Wood density

Wood density in earlywood

Wood density in latewood

Wood density

**Other**—Other traits not fitting any of the groups:

Branch age

Diameter selected trees

Genetic variability

Mean climate sensitivity of diameter increment

Mean climate sensitivity of height growth

Mycorrhizal colonization rate

Recovery to negative pointer years

Recovery to positive pointer years

Relative resilience to negative pointer years

Relative resilience to positive pointer‐ years

Resilience to negative pointer years

Resilience to positive pointer years

Resistance to negative pointer years

Resistance to positive pointer years

## Appendix C Tables of Results

**TABLE C1:** Results of all species subgroup analyses that had sufficient data for analysis.

Analysis	Trait	Subgroup	Num.obs	Prevalence	CI.lb	CI.ub	*p*	QMp	*R* ^2^	*I* ^2^
Provenance‐effect	Drought tolerance	Species	22	0.40	0.19	0.62	0**	0.69	0.00	35.02
		*A. alba*	14	0.37	0.14	0.63	0**			
		*F. sylvatica*	8	0.47	0.07	0.89	0.69			
	**Growth**	**Species**	**199**	**0.80**	**0.74**	**0.86**	**0****	**0****	**18.62**	**30.71**
		*A. alba*	14	1.00	0.85	1.00	0**			
		*B. pendula*	9	0.98	0.76	1.00	0.72			
		*F. sylvatica*	38	0.57	0.42	0.70	0**			
		*P. abies*	22	0.88	0.72	0.99	0.16			
		*P. halepensis*	26	0.76	0.58	0.92	0.03*			
		*P. nigra*	19	0.72	0.50	0.90	0.02*			
		*P. pinaster*	17	0.91	0.72	1.00	0.3			
		*P. sylvestris*	20	0.85	0.68	0.97	0.11			
		*Q. ilex*	9	0.58	0.29	0.85	0**			
		*Q. petraea*	5	1.00	0.67	1.00	0.99			
		*Q. suber*	20	0.88	0.71	0.99	0.18			
	Leaf anat./morph.	Species	28	0.55	0.36	0.73	0**	0.94	0.00	38.19
		*F. sylvatica*	14	0.54	0.27	0.80	0**			
		*Q. ilex*	8	0.50	0.14	0.86	0.87			
		*Q. suber*	6	0.59	0.19	0.94	0.82			
	**Leaf chem. comp**.	**Species**	**11**	**0.53**	**0.19**	**0.85**	**0****	**0.03***	**49.69**	**41.63**
		*F. sylvatica*	5	0.84	0.49	1.00	0**			
		*Q. suber*	6	0.23	0.00	0.63	0.03*			
	Physiology	Species	58	0.35	0.23	0.47	0**	0.51	0.98	60.73
		*F. sylvatica*	17	0.32	0.12	0.55	0**			
		*P. halepensis*	7	0.51	0.17	0.85	0.37			
		*P. pinaster*	13	0.27	0.04	0.57	0.78			
		*Q. ilex*	14	0.44	0.20	0.69	0.51			
		*Q. suber*	7	0.10	0.00	0.50	0.3			
	Survival	Species	51	0.90	0.79	0.98	0**	0.21	0.00	0.00
		*F. sylvatica*	7	0.87	0.54	1.00	0**			
		*P. halepensis*	9	0.91	0.63	1.00	0.83			
		*P. nigra*	12	0.92	0.68	1.00	0.75			
		*P. sylvestris*	9	1.00	0.88	1.00	0.17			
		*Q. suber*	14	0.72	0.46	0.93	0.44			
Provenance X Environment‐effect	Growth	Species	65	0.58	0.43	0.72	0**	0.1	20.28	39.44
		*A. alba*	11	0.68	0.30	0.98	0**			
		*F. sylvatica*	10	0.28	0.04	0.58	0.1			
		*P. abies*	8	0.81	0.44	1.00	0.6			
		*P. halepensis*	7	0.96	0.58	1.00	0.24			
		*P. nigra*	9	0.40	0.06	0.79	0.31			
		*P. pinaster*	9	0.44	0.10	0.80	0.37			
		*P. sylvestris*	11	0.62	0.29	0.91	0.82			
Survival		Species	16	0.74	0.39	0.99	0**	0.46	0.00	10.31
		*P. nigra*	9	0.63	0.16	0.99	0**			
		*P. sylvestris*	7	0.86	0.35	1.00	0.46			
Correlation Clinal‐effect	Growth	Species	52	0.20	0.11	0.30	0**	0**	36.70	85.26
		*F. sylvatica*	8	0.00	0.00	0.10	0.1			
		*P. abies*	14	0.51	0.33	0.69	0**			
		*P. halepensis*	21	0.12	0.03	0.24	0.06			
		*P. pinaster*	9	0.24	0.06	0.46	0.02*			
linear Clinal‐effect	Growth	Species	24	0.22	0.07	0.42	0**	0.03*	34.88	55.05
		*F. sylvatica*	12	0.05	0.00	0.26	0**			
		*P. sylvestris*	12	0.40	0.16	0.66	0.03*			

*Note:* Columns one to three give the type of effect, the trait group and the species that were included in the particular subgroup analysis, respectively. Rows with trait group and ‘Species’ inform of the overall analysis whereas rows with a species name inform about the specific subgroup. Num.obs is the number of observations within the analysis/the species, prevalence gives the estimated effect size of all/the respecitive species in the analysis. Likewise, CI.lb, CI.ub give the upper and lower confidence intervals of the estimated effects and *p* their *p*‐value, respectively. QMp gives the *p* value of the test for subgroup difference. The significance codes for both *p* and QMp (*p* ≤ 0.01 ≤ 0.05 or ≤ 0.1) are indicated with a “**,” “*,” or “.” respectively. *R* ^2^ gives the amount of accounted variability and *I* ^2^ the between study heterogeneity. Analyses with significant subgroup differences (*p* ≤ 0.05) and an *I* ^2^ below 50 are bold.

**TABLE C2:** Results of all regressions against age that had sufficient data for analysis.

Analysis	Trait	Num.obs	QMp	*R* ^2^	*I* ^2^
Provenance‐effect	Allocation	24	0.29	1.41	51.20
	Drought tolerance	34	0.83	0.00	11.42
	Frost tolerance	11	0.74	0.00	47.94
	Growth	214	0.07	1.89	37.40
	Leaf anat./morph.	40	0.73	0.00	0.00
	Leaf chem. comp.	36	0.62	0.00	70.21
	**Phenology**	**57**	**0.04***	**16.98**	**28.86**
	Physiology	70	0.42	0.00	63.75
	Plant morphology	42	0.71	0.00	47.47
	Reproduction	27	0.75	0.00	0.00
	Survival	71	0.1	0.00	0.00
	Wood anatomy	24	0.47	0.00	37.48
Provenance × Environment‐effect	Allocation	16	0.82	0.00	41.34
	Drought tolerance	16	0.11	8.89	45.45
	**Growth**	**96**	**0.02***	**12.94**	**48.95**
	Leaf anat./morph.	17	0.67	0.00	71.36
	Leaf chem. comp.	13	0.38	0.00	74.83
	Phenology	22	0.21	3.48	53.48
	Physiology	17	0.07	45.29	18.42
	**Plant morphology**	**23**	**0.01***	**44.05**	**27.75**
	Reproduction	16	0.38	0.00	37.41
	Survival	34	0.15	28.42	4.11
	Wood anatomy	11	0.21	14.96	48.33
Correlation Clinal‐effect	Growth	88	0.34	0.00	89.66
	Phenology	15	0.23	8.34	80.65
	Plant morphology	17	0.55	0.00	90.52
	Survival	39	0.38	0.00	66.98
	Wood anatomy	13	0.21	4.82	58.84
Correlation Transfer‐effect	Growth	18	0.06	0.46	44.39
	**Survival**	**14**	**0****	**0.00**	**0.00**

*Note:* Columns one and two give the type of effect and the trait group, respectively. Num.obs gives the number of observations within the analysis. QMp gives the *p* value of the test of moderators. The significance codes for QMp (*p* ≤ 0.01 ≤ 0.05 or ≤ 0.1) are indicated with a “**,” “*,” or “.” respectively. *R* ^2^ gives the amount of accounted variability and *I* ^2^ the between study heterogeneity. Analyses with a significant moderator test (*p* ≤ 0.05) and an *I* ^2^ below 50 are bold.
